# Tyrosine phosphatase SHP2 negatively regulates NLRP3 inflammasome activation via ANT1-dependent mitochondrial homeostasis

**DOI:** 10.1038/s41467-017-02351-0

**Published:** 2017-12-18

**Authors:** Wenjie Guo, Wen Liu, Zhen Chen, Yanhong Gu, Shuang Peng, Lihong Shen, Yan Shen, Xingqi Wang, Gen-Sheng Feng, Yang Sun, Qiang Xu

**Affiliations:** 1 State Key Laboratory of Pharmaceutical Biotechnology and Collaborative Innovation Center of Chemistry for Life Sciences, School of Life Sciences, Nanjing University, Nanjing, 210023 China; 2 Department of Diabetes Complications and Metabolism, Beckman Research Institute of City of Hope, Duarte, CA 91010 USA; 3 Department of Oncology, The First Affiliated Hospital with Nanjing Medical University, Nanjing, 210029 China; 4 Department of Pathology, and Division of Biological Sciences, University of California San Diego, La Jolla, CA 92093 USA

## Abstract

Aberrant activation of NLRP3 inflammasome has an important function in the pathogenesis of various inflammatory diseases. Although many components and mediators of inflammasome activation have been identified, how NLRP3 inflammasome is regulated to prevent excessive inflammation is unclear. Here we show NLRP3 inflammasome stimulators trigger Src homology-2 domain containing protein tyrosine phosphatase-2 (SHP2) translocation to the mitochondria, to interact with and dephosphorylate adenine nucleotide translocase 1 (ANT1), a central molecule controlling mitochondrial permeability transition. This mechanism prevents collapse of mitochondrial membrane potential and the subsequent release of mitochondrial DNA and reactive oxygen species, thus preventing hyperactivation of NLRP3 inflammasome. Ablation or inhibition of SHP2 in macrophages causes intensified NLRP3 activation, overproduction of proinflammatory cytokines IL-1β and IL-18, and increased sensitivity to peritonitis. Collectively, our data highlight that, by inhibiting ANT1 and mitochondrial dysfunction, SHP2 orchestrates an intrinsic regulatory loop to limit excessive NLRP3 inflammasome activation.

## Abstract (web summary)

The NLRP3 inflammasome is central to a variety of inflammatory diseases, but how it is regulated to prevent excessive inflammation is not clear. Here the authors show that NLRP3 activation causes SHP2 translocation to the mitochondria to interact with and dephosphorylate ANT1, thus stabilizing the mitochondria and preventing release of proinflammatory mitochondrial DNA and ROS.

## Introduction

Inflammasomes are multi-molecular signaling complexes that have a crucial function in host defense against infection, as well as various autoimmune and inflammatory disorders^1^. Inflammsomes are activated upon various cellular stresses that promote caspase-1-dependent maturation of interleukin-1β (IL-1β) and IL-18^2^. Among a number of inflammasomes identified, the Nod-like receptor family, pyrin domain containing 3 (NLRP3) inflammasome is the most extensively studied due to its robust activation by a variety of stimuli, including infection, tissue damage, and metabolic stress. Typically, activation of NLRP3 inflammasome requires two signals. The first priming signal (Signal 1), classically triggered by microbe-derived lipopolysaccharide (LPS), upregulates transcription of proinflammatory cytokines and inflammasome components via activation of transcription factor nuclear factor-κB (NF-κB). The second activation signal (Signal 2), stimulated by adenosine triphosphate (ATP), monosodium urate (MSU) and nigericin, assembles cytosolic inflammasome components, resulting in cleavage of caspase-1 and production of pro-inflammatory cytokines, i.e., IL-1β or IL-18^3^. Mitochondrial dysfunction, exemplified by the mitochondrial permeability transition, overproduction of reactive oxygen species (ROS), and the resultant release of mitochondrial DNA, is crucial for the Signal 2 activation of NLRP3 inflammasome^4–6^. Given the importance of NLRP3 inflammasome in the pathogenesis of many inflammatory diseases such as peritonitis, multiple sclerosis and obesity^7–9^, understanding the positive and negative regulation of NLRP3 inflammasome may provide insight into pathology and identify new therapeutic strategies.

Src homology 2 (SH2) domain-containing tyrosine phosphatase-2 (SHP2) is a ubiquitously expressed non-receptor protein tyrosine phosphatase (PTP). Encoded by the *PTPN11* gene in humans, SHP2 protein consists N-SH2 and C-SH2 domains, both of which are important for its subcellular localization, and a PTP domain, which is crucial for its enzymatic activity^10^. SHP2 has been identified to have critical functions in cell proliferation and differentiation in response to growth factors and cytokines^11^, such as epidermal growth factor and platelet-derived growth factor-induced Ras-Raf-Erk cascade^12–14^. Increasing evidence indicates that SHP2 is also involved in immune signaling and inflammatory response. For example, SHP2 has been shown to negatively regulate TLR3-activated and TLR4-activated interferon (IFN)-β production in macrophages^15^. However, whether SHP2 has a regulatory function in NLRP3 inflammasome, the key effector of innate immune response, has not been investigated.

Adenine nucleotide translocase 1 (ANT1) is an ADP/ATP translocase located in the inner mitochondrial membrane. Protein complex comprised ANT1, voltage-dependent anion channel, and cyclophilin D has a crucial function in the maintenance of mitochondrial membrane potential and permeability^16,17^. Mice with deactivated heart/muscle isoform of ANT1 have characteristics of myopathy and cardiomyopathy with a severe defect in mitochondria-coupled respiration^18^. Phosphorylation of ANT1 by the Src family kinase members Src and Lck has been shown to be critical for mitochondrial bioenergetics and cardioprotection^19,20^. Given the control of ANT1 in mitochondrial homeostasis, we hypothesize that the dysregulation of ANT1 is an underlying mechanism of NLRP3 inflammasome overactivation.

In this study, we investigate the role of SHP2 in NLRP3 inflammasome activation and its implication in inflammatory diseases. By using macrophage-specific conditional SHP2 knockout (cSHP2-KO) mouse, we demonstrate that SHP2 is a negative regulator of NLRP3 inflammasome. Furthermore, we identify ANT1 as phosphatase substrate of SHP2 upon its translocation to mitochondria, which mediates the negative regulation of NLRP3 inflammasome by SHP2. Specifically, SHP2-mediated dephosphorylation of ANT1 at Tyr 191 is essential for mitochondrial homeostasis and mitigation of NLRP3 inflammasome activation. Collectively, our findings provide new insights into the dynamic regulation of NLRP3 inflammasome activation through a SHP2-ANT1-mediated negative regulatory loop.

## Results

### SHP2 inhibits NLRP3 inflammasome activation in macrophages

**Fig. 1:**
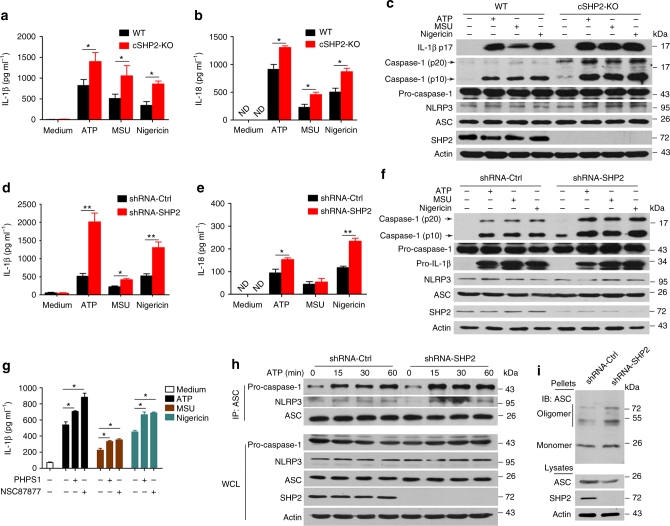
SHP2 deficiency results in excessive activation of NLRP3 inflammsome in macrophages. Two groups of macrophages, i.e., peritoneal macrophages from conditional SHP2 knockout (cSHP2-KO) and wild-type (WT) mice **a–c**, or PMA-differentiated THP-1 cells with shRNA-Ctrl or shRNA-SHP2 lentivirus **d**, **f**, were primed with 100 ng ml^−1^ LPS for 3 h, and then stimulated with ATP (5 mM, 1 h), MSU (500 µg ml^−1^, 2 h) or Nigericin (10 µM, 2 h), respectively. **a**, **b**, **d**, **e** Enzyme-linked immunosorbent assay (ELISA) of IL-1β and IL-18 in culture supernatants. ND represents not detectable. **c**, **f** Immunoblot analysis of cell lysates. **g** ELISA of IL-1β in supernatants of THP-1-derived macrophages left untreated or treated with SHP2 inhibitor PHPS1 (10 μM) or NSC87877 (10 μM) for 1 h, followed by LPS treatment and ATP, MSU or Nigericin stimulation. **h** Immunoblot analysis of proteins immunoprecipitated with anti-ASC from lysates of ATP-treated SHP2 knockdown THP-1-derived macrophages. **i** Immunoblot analysis of ASC in cross-linked pellets (upper panels) and cell lysates (lower panels) from ATP-treated SHP2 knockdown THP-1-derived macrophages. *P < 0.05, **P < 0.01, one-way ANOVA for multiple comparisons. Data are representative of three independent experiments (mean and SEM of three independent samples in **a**, **b**, **d**, **e**, **g**)

To examine the function of SHP2 in NLRP3 inflammasome activation, we generated macrophage-specific (Lyz2-Cre) cSHP2-KO mice (Supplementary Fig. 1). In primary peritoneal macrophages isolated from cSHP2-KO mice, NLRP3 inflammasome activation by ATP, MSU, or Nigericin was remarkably intensified, evidenced by increased caspase-1 cleavage, as well as over-production of IL-1β and IL-18 (Fig. 1a–c). To confirm this result in human cells, we also stimulated NLRP3 inflammasome activation in THP-1-derived macrophages. Consistently, SHP2 knockdown significantly augmented NLRP3 inflammasome activation (Fig. 1d-f). In addition, pharmacological inhibition of SHP2 with NSC87877 or PHPS1 resulted in a similar potentiation of IL-1β production (Fig. 1g). As the hallmark of Signal 2, the assembly of NLRP3/ASC (apoptosis-associated speck-like protein containing a CARD)/pro-caspase-1 complex was also enhanced when SHP2 was knocked down (Fig. 1h). Furthermore, ATP-stimulated ASC oligomerization was higher in cells with SHP2 knockdown, when compared with that in cells transfected with control short hairpin RNA (shRNA) (Fig. 1i). Collectively, these observations suggest that SHP2 negatively regulates NLRP3 inflammasome activation in the macrophage.

### SHP2 inhibits NLRP3 inflammasome activation in vivo

**Fig. 2:**
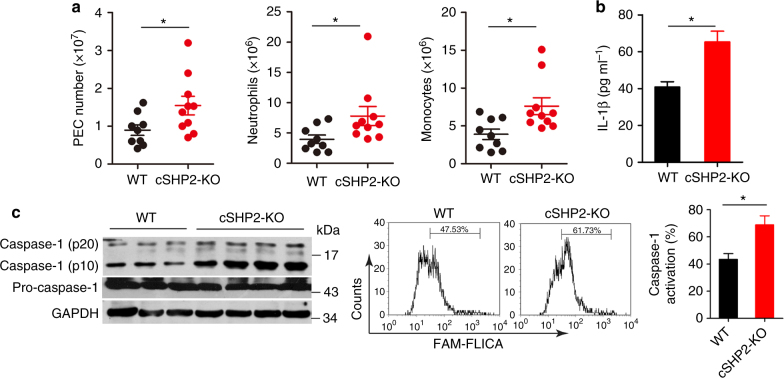
SHP2 deficiency in macrophages aggravates murine peritonitis model. **a**–**c** Eight-week-old female cSHP2-KO and WT mice were killed 12 h after Alum injection and peritoneal cavities were washed with PBS. **a** Flow cytometry analysis of peritoneal exudate cells (PECs) 12 h after Alum injection in mice. **b** ELISA of IL-1β level in the lavage fluid 8 h after Alum injection in mice. **c** Immunoblot analysis (left panel) and flow cytometry analysis (right panel) of caspase-1 activation in PECs 12 h after Alum injection in mice. Data are representative of three independent experiments (mean and SEM of 10 mice per group), *P < 0.05 by Student’s *t*-test

Given that SHP2 inhibits NLRP3 inflammasome activation in vitro, we further examined the role of SHP2 in the alum-induced murine peritonitis model. Upon alum challenge, cSHP2-KO mice had higher numbers of total peritoneal exudate cells (PECs), neutrophils, and monocytes, when compared with the wild-type (WT) littermates (Fig. 2a and Supplementary Fig. 2). Such pro-inflammatory profile in cSHP2-KO mice was also associated with a significantly higher level of IL-1β in the lavage fluid (Fig. 2b). Consistently, caspase-1 activation was more evident in PECs from cSHP2-KO mice compared with WT controls (Fig. 2c). These findings demonstrate that SHP2 inhibits NLRP3 inflammasome activation in vivo, and its deficiency leads to overactivation of NLRP3 inflammasome in the context of inflammatory diseases.

### SHP2 deficiency leads to mitochondrial dysfunction

**Fig. 3:**
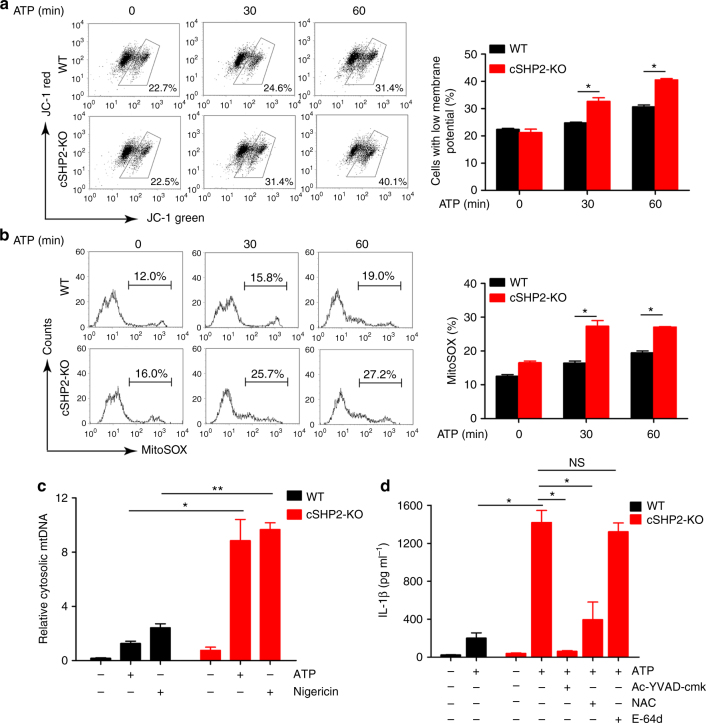
SHP2 deficiency leads to mitochondrial dysfunction and excessive NLRP3 inflammasome activation. **a**, **b** Flow cytometry analysis of mitochondrial membrane potential by JC-1 staining **a** or mitochondrial ROS by MitoSOX staining **b** in peritoneal macrophages from conditional SHP2 knockout (cSHP2-KO) and wild-type (WT) mice, and left untreated or treated with ATP (5 mM) for indicated times. **c** Quantitative real-time PCR analysis of mtDNA released from peritoneal macrophages from cSHP2-KO and WT mice and left unstimulated (medium) or primed with LPS (100 ng ml^−1^) for 3 h and stimulated with ATP (5 mM, 1 h) and Nigericin (10 µM, 2 h). **d** ELISA of IL-1β in supernatants of peritoneal macrophages from cSHP2-KO and WT mice, which were primed with LPS (100 ng ml^−1^) for 3 h, and left untreated or treated with Ac-YVAD-cmk (30 μM), NAC (5 mM), or E-64d (20 μM) for 1 h, followed by stimulation of ATP (5 mM) for 1 h. *P < 0.05, **P < 0.01, one-way ANOVA for multiple comparisons; NS represents no significance. Data are presented as mean ± SEM of three independent experiments in **a**–**d**

Our data from Figs. 1 and 2 indicate that the Signal 2 of inflammasome activation is augmented in macrophages and mice with SHP2 deficiency, evidenced by increased level of caspase-1 cleavage but not pro-caspase-1. We thus examined the role of SHP2 in the mitochondrial dysfunction, which subsequently contributes to NLRP3 inflammasome activation. ATP stimulation of primary peritoneal macrophages isolated from WT mice caused membrane potential collapse (JC-1 staining), mtROS production (mitochondrial reactive oxygen species, MitoSOX staining), and mitochondrial DNA (mtDNA) release to the cytosol, which were potentiated in macrophages isolated from cSHP2-KO mice (Fig. 3a–c). Similarly, membrane potential collapse, mtROS production and mtDNA release to cytosol were also aggravated in THP-1-derived macrophages with SHP2 silencing (Supplementary Fig. 3). Of note, we did not observe any significant change in cell survival or lysosome activity due to SHP2 knockdown (Supplementary Fig. 4). In addition, the augmented IL-1β production in SHP2-knockdown THP-1-derived macrophages in response to ATP was abrogated by either ROS scavenger NAC (N-acetylcysteine) or caspase-1 inhibitor Ac-YVAD-cmk, but was marginally affected by E-64d, a lysosome inhibitor (Fig. 3d and Supplementary Fig. 3d), suggesting ROS release and caspase-1 activation are required for the excessive activation of NLRP3 inflammasome due to SHP2 deficiency.

### SHP2 translocates into mitochondria and interacts with ANT1

**Fig. 4:**
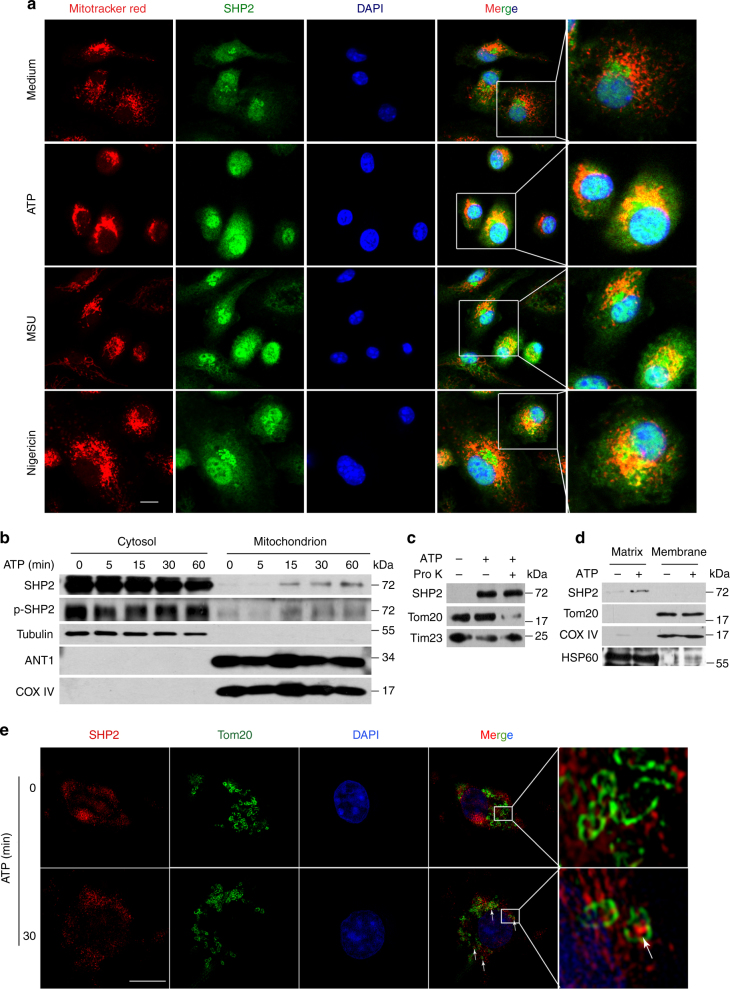
SHP2 translocates into mitochondrial matrix during NLRP3 inflammasome activation. **a** Immunofluorescence analysis of SHP2 and mitochondria from bone marrow-derived macrophages with untreated (medium) or ATP (5 mM, 15 min), or MSU (500 µg ml^−1^, 2 h) or Nigericin (10 µM, 2 h) treatment. Scale bar, 5 µm. **b** Immunoblot analysis of mitochondrial and cytosolic components of THP-1-derived macrophages treated with ATP (5 mM) for indicated times. **c** Immunoblot analysis of SHP2 location in mitochondria from THP-1-derived macrophages. Cells were treated with 5 mM ATP for 30 min, then mitochondria were isolated and incubated with 40 μM proteinase K for 30 min. Tom20 in mitochondrial outer membrane (MOM) and Tim23 in mitochondrial inner membrane (MIM) were used as controls, respectively. **d** Immunoblot analysis of SHP2 expression in submitochondrial fractions from THP-1-derived macrophages treated with ATP (5 mM, 30 min). Tom20, COX IV, and HSP60 were used to represent MOM, MIM, and mitochondrial matrix protein, respectively. **e** Immunofluorescence analysis of SHP2 and Tom20 from bone marrow-derived macrophages with untreated (medium) or ATP (5 mM, 30 min) by structured-illumination microscopy (SIM). Scale bar, 5 µm. Data are representative of three independent experiments

Next, we aimed to elucidate the mechanism by which SHP2 controls mitochondrial permeability. Immunostaining of SHP2 hinted that SHP2 could translocate from cytosol to mitochondria upon ATP, MSU or Nigericin treatment (Fig. 4a). Consistently, immunoblot analysis of mitochondrial and cytosolic fractions confirmed this phenomenon (Fig. 4b). Considering that SHP2 translocate to mitochondria, another question was raised. Where was SHP2 located in mitochondria? To answer this issue, we used proteinase K to digest mitochondrial outer membrane (MOM) proteins in the mitochondrial fraction isolated from THP-1 cells. Protein component of MOM, e.g., Tom20 was digested while SHP2 and Tim23, a mitochondrial inner membrane (MIM) were protected from the degradation in mitochondrial fraction isolated from the ATP-treated cells (Fig. 4c), suggesting that SHP2 is not located in the MOM. By using alkaline extraction of mitochondrial matrix^21^, we found that SHP2 may translocate to the matrix of mitochondria (Fig. 4d). This mitochondrial localization of SHP2 was also observed by structured-illumination microscopy (Deltavision, OMX. GE). Tom20 in MOM was stained and indicated a tube-like shape of mitochondria (Fig. 4e). SHP2 was located in this tube-like shape under ATP-stimulation compared with unstimulated cells (Figs. 4e, and 3D-reconstitution image in Supplementary Movie 1 and 2).

**Fig. 5:**
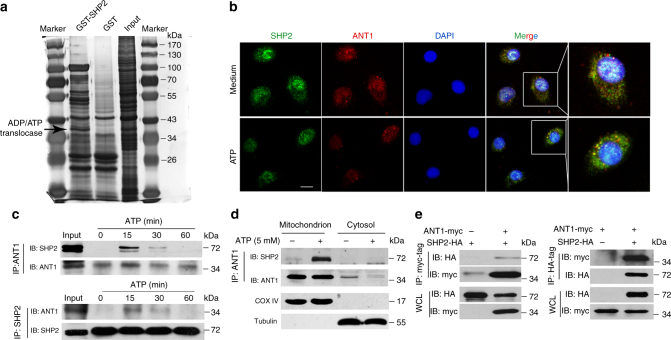
SHP2 interacts with ANT1 during NLRP3 inflammasome activation. **a** Sliver staining of GST pull-down proteins using GST-SHP2 fusion protein. The proteins interacting with GST-SHP2 were identified by mass spectrometry. **b** Immunofluorescence analysis of SHP2 and ANT1 from bone marrow-derived macrophages with untreated (medium) or ATP (5 mM, 15 min) treatment. Scale bar, 5 µm. **c** Immunoblot analysis of reciprocal co-immunoprecipitation (Co-IP) from THP-1-derived macrophages treated with ATP (5 mM) for indicated times. **d** Immunoblot analysis of Co-IP from mitochondrial and cytosolic components in THP-1-derived macrophages treated with ATP for 15 min. **e** Immunoblot analysis of reciprocal Co-IP from HEK293T cells overexpressing HA-tagged SHP2 and myc-tagged ANT1. Data are representative of three independent experiments

Furthermore, we examined the SHP2-interacting proteins using GST-pulldown assay, using GST-SHP2 as a bait. Mass spectrometry profiling of binding protein partners of SHP2 identified ANT1 as a high-confidence hit (Fig. 5a and Supplementary Table 1). This observation motivated us to further investigate whether SHP2 indeed interacts with ANT1 during NLRP3 inflammasome activation. The confocal immunofluorescence analysis showed that SHP2 co-localized with ANT1 upon ATP treatment (Fig. 5b), providing an evidence that SHP2 and ANT1 are interacting with each other during NLRP3 inflammasome activation. Moreover, reciprocal co-immunoprecipitation assay proved the endogenous association between SHP2 and ANT1 in THP-1-derived macrophages treated with ATP for 15–30 min (Fig. 5c). Interestingly, the assembly of NLRP3/ASC/pro-caspase-1 complex was detected as early as 5 min upon ATP treatment (Supplementary Fig. 5). More importantly, these two partners interplayed in the mitochondria when NLRP3 inflammasome was activated (Fig. 5d). In addition, we confirmed this result in HEK293T cells, where we detected the interaction between the exogenous HA-tagged SHP2 and myc-tagged ANT1 (Fig. 5e).

**Fig. 6:**
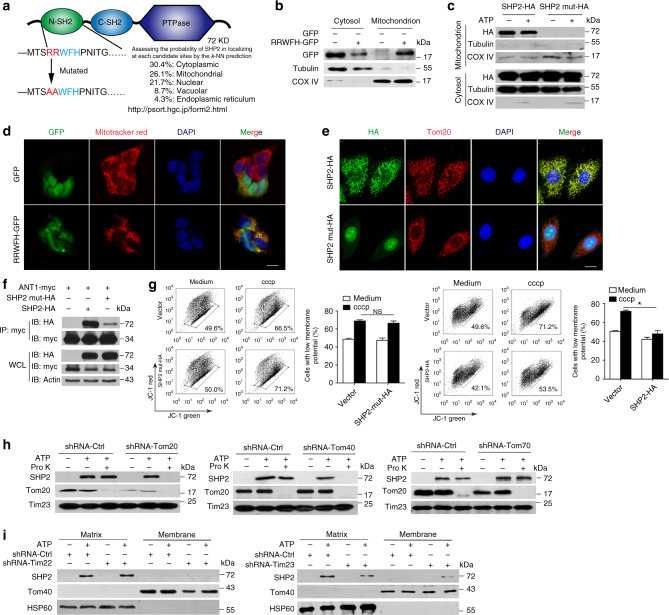
Tom20/Tom40 and Tim23 complex are necessary for SHP2 translocation to mitochondrial matrix. **a** Predication of mitochondrial target sequence in SHP2 by PSORT II. **b** Immunoblot analysis of GFP localization in submitochondrial fractions from HEK293T cells which were transfected with GFP or RRWFH-GFP plasmid. **c** Immunoblot analysis of SHP2 localization in submitochondrial fractions from HEK293T cells which were transfected with SHP2-HA or SHP2-mut-HA (mitochondrial target sequence mutation, RRWFH mutated to AAWFH) plasmid followed by ATP treatment (5 mM, 30 min). **d** Immunofluorescence analysis mitochondrial localization of GFP-tagged RRWFH motif in HEK293T cells. Scale bar, 10 µm. **e** Immunofluorescence analysis mitochondrial localization of SHP2-HA or SHP2-mut-HA plasmid in HEK293T cells. Scale bar, 10 µm. **f** Co-immunoprecipitation (Co-IP) analysis of the interaction of SHP2 and ANT1 in HEK293T cells, which were transfected with ANT1-myc and SHP2-HA or SHP2-mut-HA. **g** Flow cytometry analysis of mitochondrial membrane potential by JC-1 staining in HEK293T cells which were transfected with SHP2-HA or SHP2-mut-HA plasmid followed by cccp treatment (20 µM, 1 h). **h** Immunoblot analysis of SHP2 in mitochondria after Tom20 or Tom40, or Tom70 sliencing. Endogenous Toms were separately knocked down by its corresponding shRNAs in THP-1 cells followed by ATP treatment (5 mM, 30 min), then mitochondria were isolated and incubated with 40 μM proteinase K (Pro K) for 30 min. Tom20 in mitochondrial outer membrane (MOM) and Tim23 in mitochondrial inner membrane (MIM) were used as control, respectively. **i** Immunoblot analysis of SHP2 expression in submitochondrial fractions from THP-1-derived macrophages treated with ATP (5 mM, 30 min) after Tim22 or Tim23 silencing. *P < 0.05 by Student’s *t*-test, NS represents no significance. Data are representative of three independent experiments (mean and SEM of three independent samples in **g**)

Given the translocation of SHP2 to mitochondria upon NLRP3 inflammasome activation, we searched for a mitochondria-targeting sequence in SHP2 protein using PSORT II prediction tool^22^. Indeed, there was RRWFH motif (Argnine-Argnine-Tryptophane-Phenylalanine-Histidine) in N-SH2 domain of SHP2 (Fig. 6a). To validate this prediction, we overexpressed the RRWFH motif tagged with green fluorescent protein (GFP) in HEK293T cells. As a result, GFP had no specific location while RRWFH-GFP predominantly localized in the mitochondria (Fig. 6b, d). When RRWFH motif was mutated, both SHP2 translocation to mitochondria and the SHP2-ANT1 interaction were diminished (Fig. 6c, e, f). As a result, this SHP2 mutant did not exert inhibitory effect on the collapse of mitochondrial membrane potential (Fig. 6g). Furthermore, to illuminate how SHP2 translocate to the matrix of mitochondria, we examined the mitochondrial translocation of SHP2 in THP-1 cells with Tom20 or Tom40, or Tom70 knockdown, as well as Tim22 or Tim23 knockdown (Supplementary Fig. 6), which mediated the protein crossed MOM and MIM into matrix after targeting to the mitochondria^23^. We found that the translocation of SHP2 into mitochondria induced by ATP treatment was almost completely blocked when Tom20 or Tom40 was knocked down, respectively (Fig. 6h). Moreover, localization of SHP2 in matrix triggered by ATP treatment was remarkably reduced by Tim23 but not Tim22 knockdown (Fig. 6i). These data indicate that Tom20 and Tom40 in MOM, as well as Tim23 in MIM are responsible for the translocation of cytosolic SHP2 into mitochondrial matrix during NLRP3 inflammasome activation. Taken together, these results suggest that during the activation of NLRP3 inflammasome, SHP2 translocates from cytosol to mitochondrial matrix and interacts with ANT1.

### ANT1 knockdown suppresses NLRP3 inflammasome activation

**Fig. 7:**
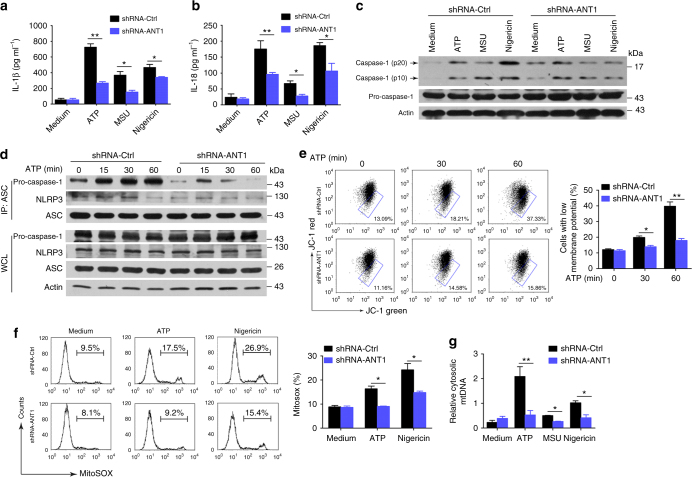
ANT1 knockdown suppresses activation of NLRP3 inflammasome. Two groups of THP-1-derived macrophages, i.e., with shRNA-Ctrl or shRNA-ANT1, were primed with 100 ng ml^−1^ LPS for 3 h, and then stimulated with ATP (5 mM, 1 h), MSU (500 µg ml^−1^, 2 h), or Nigericin (10 µM, 2 h), respectively. **a**, **b** ELISA of IL-1β and IL-18 in the culture supernatant. **c** Immunoblot analysis of cell lysates from THP-1-derived macrophages. **d** Immunoblot analysis of Co-IP from THP-1-derived macrophages. **e**, **f** Flow cytometry analysis of mitochondrial membrane potential by JC-1 staining **e** or mitochondrial ROS by MitoSOX staining **f** from THP-1-derived macrophages with shRNA-Ctrl or shRNA-ANT1 lentivirus, followed by LPS treatment and ATP or Nigericin stimulation. **g** Quantitative real-time PCR analysis of mtDNA released from ANT1-knockdown THP-1-derived macrophages and left unstimulated (medium) or primed with LPS and stimulated with ATP, MSU or Nigericin. *P < 0.05, **P < 0.01, one-way ANOVA for multiple comparisons. Data are representative of three independent experiments (mean and SEM of three independent samples in **a**, **b**, **e–g**)

The interaction of SHP2 and ANT1 prompted us to determine the role of ANT1 in mitochondrial permeabilization and NLRP3 inflammasome activation. We knocked down ANT1 in THP-1-derived macrophages with stable transfection of ANT1 shRNA and investigated the consequence in terms of inflammasome activation. Compared with control shRNA group, we found that in ANT1-knockdown THP-1-derived macrophages, (1) secretions of IL-1β and IL-18, and the cleavage of pro-caspase-1 were significantly inhibited (Fig. 7a–c); (2) the NLRP3/ASC/pro-caspase-1 assembly was profoundly inhibited (Fig. 7d); (3) ATP-induced collapse of mitochondrial membrane potential was attenuated (Fig. 7e); and (4) various danger signal-stimulated release of mtROS and mtDNA were almost abolished (Fig. 7f,g). Conversely, overexpression of ANT1 markedly enhanced mitochondrial permeabilization (Supplementary Fig. 7). These findings indicate that ANT1 is required for mitochondrial permeability transition and the subsequent NLRP3 inflammasome activation.

### SHP2 inhibits NLRP3 activation in an ANT1-dependent manner

**Fig. 8:**
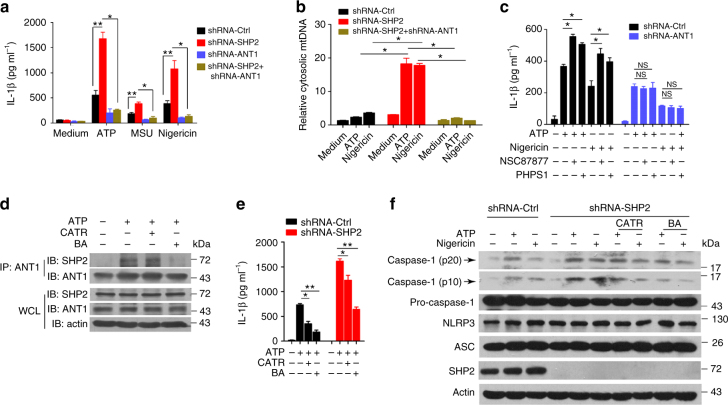
SHP2 inhibits NLRP3 inflammasome activation in an ANT1-dependent manner. **a**, **b** SHP2 knockdown, ANT1 knockdown and SHP2-ANT1 double knockdown THP-1-derived macrophages were primed with 100 ng ml^−1^ LPS for 3 h, followed by ATP (5 mM, 1 h), MSU (500 µg ml^−1^, 2 h), or Nigericin (10 µM, 2 h) stimulation, respectively. **a** ELISA of IL-1β in the supernatant. **b** Quantitative real-time PCR analysis of mtDNA. **c** ELISA of IL-1β in the culture supernatant from ANT1 knockdown THP-1-derived macrophages and left untreated or treated with NSC87877 (10 μM) or PHPS1 (10 μM) for 1 h, followed by ATP or Nigericin stimulation. **d**–**f** LPS-primed SHP2 knockdown THP-1-derived macrophages were treated with CATR (5 mM) or BA (50 µM) for 1 h, followed by ATP or Nigericin stimulation. **d** Immunoblot analysis of Co-IP from THP-1-derived macrophages treated with CATR or BA. **e** ELISA of IL-1β in the culture supernatant. **f** Immunoblot analysis of cell lysates from THP-1-derived macrophages treated with CATR or BA. *P < 0.05, **P < 0.01, one-way ANOVA for multiple comparisons, NS represents no significance. Data are representative of three independent experiments (mean and SEM of three independent samples in **a**–**c**, **e**)

The interaction between SHP2 and ANT1 (Fig. 5), and the opposite effects of SHP2 and ANT1 shRNAs in the activation of NLRP3 inflammasome (Figs. 1 and 7) raised the possibility that the SHP2 may inhibit ANT1 to suppress NLRP3 inflammasome activation. To test this hypothesis, we compared the effects of SHP2 only, ANT1 only, and both SHP2 and ANT1 knockdown in THP-1-derived macrophages (Supplementary Fig. 6). Remarkably, the overactivation of NLRP3 inflammasome (hallmarked by the excessive production of IL-1β) and damaged mitochondria (signified by cytosolic mtDNA release) resulted from SHP2 knockdown was completely reversed by ANT1 and SHP2 double knockdown (Fig. 8a, b), which is similar to that of ANT1 only knockdown (Fig. 7a, g). Similarly, the effect of SHP2 inhibitors, NSC87877 and PHPS1, in enhancing IL-1β production induced by ATP or Nigericin was abrogated in THP-1-derived macrophages with ANT1 knockdown (Fig. 8c). Furthermore, ANT1 specific inhibitor bongkrekic acid (BA) and CATR (Carboxyatractyloside) interrupted the interaction between SHP2 and ANT1 (Fig. 8d), and also reversed SHP2 deficiency-induced IL-1β production (Fig. 8e) and caspase-1 activation (Fig. 8f). These data indicated that SHP2 negatively regulates NLRP3 inflammasome activation in an ANT1-dependent manner.

### SHP2-ANT1 interaction mediates mitochondrial homeostasis

**Fig. 9:**
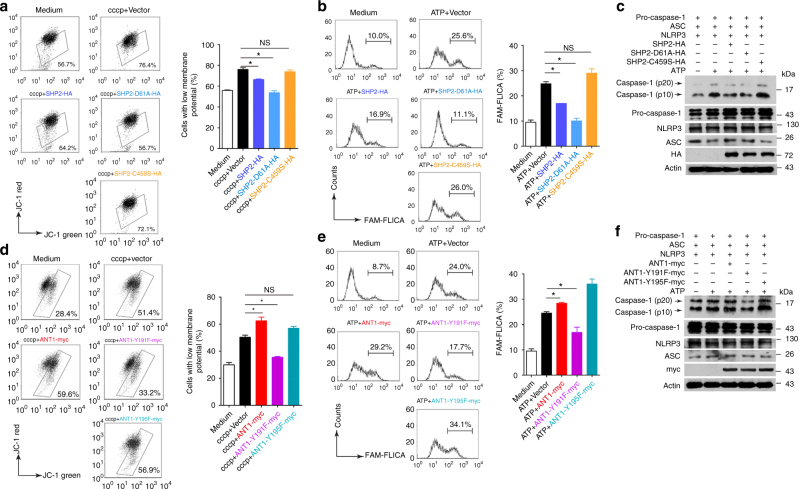
SHP2 dephosphorylation of ANT1 at Tyr 191 is essential for mitochondrial homeostasis. **a** Flow cytometry analysis of mitochondrial membrane potential by JC-1 staining of HEK293T cells overexpressing Vector, SHP2-HA, SHP2-D61A-HA, or SHP2-C459S-HA plasmid and left untreated (medium) or treated with cccp (20 µM, 1 h). **b**,**c** HEK293T cells were transfected with pro-caspase-1, ASC, NLRP3, and SHP2-HA, SHP2-D61A-HA, or SHP2-C459S-HA plasmid, respectively, followed by ATP (5 mM, 1 h) treatment. **b** Flow cytometry analysis of caspase-1 activation. **c** Immunoblot analysis of caspase-1 activation. **d** Flow cytometry analysis of mitochondrial membrane potential by JC-1 staining of HEK293T cells overexpressing Vector, ANT1-myc, ANT1-Y191F-myc, or ANT1-Y195F-myc plasmid and left untreated (medium) or treated with cccp (20 µM, 1 h). **e**, **f** HEK293T cells were transfected with pro-caspase-1, ASC, NLRP3, and ANT1-myc, ANT1-Y191F-myc or ANT1-Y195F-myc plasmid respectively followed by ATP (5 mM, 1 h) treatment. **e** Flow cytometry analysis of caspase-1 activation. **f** Immunoblot analysis of caspase-1 activation. *P < 0.05, one-way ANOVA for multiple comparisons, NS represents no significance. Data are representative of three independent experiments (mean and SEM of three independent samples in **a**, **b**, **d**, **e**)

**Fig. 10:**
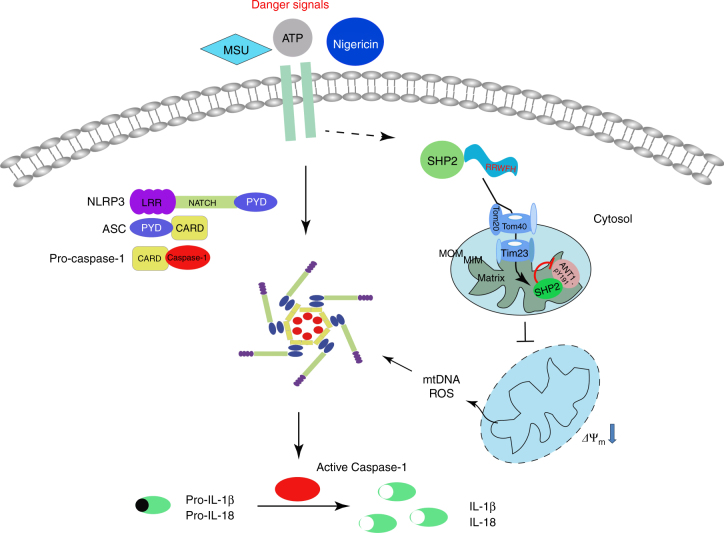
The graphic illustration of the mechanism of SHP2 regulating NLRP3 inflammasome activation. Stimulated by Signal 2 activators (e.g., ATP, MSU, and Nigericin), SHP2 is recruited to mitochondria through its RRWFH motif. With the help of Tom20/Tom40 and Tim23 complex, SHP2 translocates into the mitochondrial matrix and dephosphorylates ANT1 at Tyr 191. This serves as a key mechanism controlling mitochondrial homeostasis, preventing leakage of mitochondrial DNA (mtDNA) and overproduction of reactive oxygen species (ROS), which results in negative regulation of NLRP3 inflammasome activation

Finally, we sought to determine whether the tyrosine phosphatase activity of SHP2 is involved in ANT1-dependent NLRP3 inflammasome regulation. We first overexpressed WT SHP2, or SHP2-D61A (Asp-61 mutated to Ala in SH2 domain, gain-of-function mutant), or SHP2-C459S (Cys-457 mutated to Ser in PTP domain, loss-of-function mutant) in HEK293T cells. As shown in Fig. 9, overexpression of WT SHP2 or SHP2-D61A but not SHP2-C459S inhibited ATP-induced mitochondrial membrane potential collapse (Fig. 9a) as well as caspase-1 activation (Fig. 9b, c), suggesting the phosphatase activity of SHP2 is required for mitochondrial homeostasis and attenuation of NLRP3 inflammasome activation. Indeed, SHP2 phosphorylation level in mitochondria was increased by ATP treatment in a time-dependent manner (Fig. 4c), indicating activation of SHP2 in mitochondria. When SHP2 was knockdown by shRNA, total tyrosine phosphorylation of ANT1 was markedly increased, suggesting that SHP2 may dephosphorylate ANT1 (Supplementary Fig. 8). To pin down the specific ANT1-interacting domain in SHP2, plasmids containing only PTP domain (SHP2-ΔSH2-HA) or only SH2 domain (SHP2-ΔPTP-HA) of SHP2 were overexpressed in HEK293T cells. Co-immunoprecipitation assay showed that SH2 domain of SHP2 interacted with ANT1 (Supplementary Fig. 9). To further determine which tyrosine phosphorylation site of ANT1 is dephosphorylated by SHP2, we created the dephosphorylated mutant of ANT1, in which tyrosine was replaced with phenylalanine (Y to F mutation). As a result, Tyr 191 but not Tyr 195 mutation of ANT1 attenuated the collapse of mitochondrial membrane potential (Fig. 9d) and suppressed caspase-1 activation (Fig. 9e, f). Noticeably, these effects were similar to those of ANT1 knockdown (Fig. 7). The data presented in Fig. 9 suggest that SHP2-mediated dephosphorylation of ANT1 at Tyr 191 is critical for mitochondrial homeostasis and attenuation of NLRP3 inflammasome activation. Collectively, these data demonstrate that SHP2, by dephosphorylating ANT1 and maintaining mitochondrial homeostasis, constitutes an intrinsic negative regulatory loop to limit NLRP3 inflammasome overactivation (Fig. 10).

## Discussion

In this study, we identified SHP2 as a negative regulator of NLRP3 inflammasome activation. Summarized in Fig. 10, NLRP3 activation triggers SHP2 translocation from cytoplasm to mitochondria, where it interacts with and dephosphorylates ANT1, a central molecule controlling mitochondrial permeability transition. These molecular events constitute a checkpoint that prevent mitochondrial damage and thereby inhibit overactivation of NLRP3 inflammasome and the consequent overproduction of proinflammatory cytokines IL-1β and IL-18. Loss of SHP2 in macrophage leads to excessive inflammasome activation in murine peritonitis model. Together our findings reveal a novel mechanism by which SHP2, through dephosphorylating ANT1, provides a crucial negative regulatory loop to prevent uncontrolled activation of NLRP3 inflammasome.

As mechanisms leading to inflammasome activation continue to be intensively investigated, several negative regulators have been identified to attenuate NLRP3 inflammasome signaling through different mechanisms. At the molecular level, leucine-rich repeat Fli-I-interacting protein 2^24^, A20^25^, small hetero dimer partner^26^ and aryl hydrocarbon receptor^27^ have been demonstrated to inhibit NLRP3 inflammasome activation in macrophage. Recently, lipin-2 has been reported to regulate P2X7 receptor sensitization to limit overactivation of NLRP3 inflammasome, which may provide clues to better understand the molecular features that characterize the high IL-1β production found in Majeed syndrome patients with *LPIN2* mutations^28^. At subcellular level, autophagy, the regulated process that allows the orderly degradation and recycling of cellular components, can preserve the mitochondrial integrity and consequently inhibits the mtDNA-activated NLRP3 inflammasome^5^. Also, NF-κB-p62-mitophagy axis has also been confirmed to restrain NLRP3 inflammasome activation, serving as a self-limiting loop for NF-κB-mediated inflammation^29^. In the present study, we found that Signal 2 stimuli (i.e., ATP, MSU, and Nigericin), whereas instigating NLRP3 activation on one hand also trigger translocation of SHP2 into mitochondria, leading to subsequent dephosphorylation of ANT1 at Tyr 191 and suppression of mitochondrial permeability transition, thus hampering NLRP3 inflammasome activation on the other hand. Notably, we detected the assembly of NLRP3/ASC/pro-caspase-1 complex as early as 5 min upon ATP treatment (Supplementary Fig. 5). This precedes the mitochondrial translocation of SHP2 and the dephosphorylation of ANT1, both of which were observed at 15–30 min (Figs. 4b and 5c). The temporal order of signal transduction suggests that under physiological conditions, the initial activation of NLRP3 inflammasome (i.e., Signal 2), while induces a burst of production of pro-inflammatory cytokines IL-1β and IL-18, also rapidly switches on a negative loop mediated by SHP2. Specifically, SHP2 is rapidly mobilized to mitochondria and suppresses ANT1-propagated mitochondrial damage, including collapse of mitochondrial membrane potential, mitochondrial permeability transition, and overproduction of mitochondrial DNA and ROS. When SHP2 was inhibited in macrophages, the negative regulatory loop became compromised or abrogated, and therefore led to uncontrolled activation of inflammasome, the extensive damage of mitochondria and unrestrained release of mitochondrial DNA and ROS, which further augment the inflammasome cascade, leading to aberrant innate immune activation (Figs. 1 and 3). In support of this notion, mice with SHP2 deficiency developed more severe sterile inflammation, signified by aggravated peritonitis (Fig. 2). Our data are in line with the previous study^15^ demonstrating the negative regulation by SHP2 in TLR3- and TLR4-activated production of pro-inflammatory cytokines such as IFN-β, IL-6, and tumor necrosis factor-α. Although we focused on the role of SHP2 in NLRP3 inflammasome activation in macrophages in the context of sterile inflammation, SHP2 may also be implicated a variety of pathogen-associated molecular patterns and other danger-associated molecular patterns-induced innate immune response in a range of immune cells. For example, SHP2 is also required for the host defense against fungal pathogens and the associated production of pro-inflammatory cytokines and chemokines including IL-1β^30.^ Future study is warranted to elucidate the comprehensive regulation of SHP2 in the innate immune response.

As a protein phosphatase, the substrates and the associated molecular functions of SHP2 largely depend upon the upstream signals and its subcellular localization^31^. In the cytosol, SHP2 can dephosphorylate Gab1, Paxillin or Sprouty proteins^32–36^, all of which are involved in the innate immune and inflammatory response. In the nucleus, SHP2 dephosphorylates STAT1 to inhibit its transcriptional activity^37^ or interacts with STAT5 to regulate prolactin-mediated signaling^38^. In the mitochondria, the role of SHP2 remains unclear. Previous studies in the brain and endothelial cells suggested that SHP2 can reduce endogenous mitochondrial ROS formation although the mechanisms are unclear^39,40^. This is in line with our finding that mitochondria-derived ROS was elevated in macrophages with SHP2 knockdown, which was associated with decreased mitochondrial membrane potential and increased cytosolic mtDNA (Fig. 3). We identified in the current study a mitochondria-targeting motif RRWFH in the N-SH2 domain of SHP2 is required for its translocation from cytosol to mitochondria and subsequent interaction with ANT1 (Fig. 6b–f). In order to arrive in the mitochondrial innermost space—the matrix, SHP2 needs to cross two membranes, the MOM and MIM^41^. Our results showed that this process was mediated by two translocase complexes, the Tom20/Tom40 complex in the MOM (Fig. 6h), as well as the Tim23 complex in the MIM (Fig. 6i). Based on mitochondria-targeting RRWFH motif, with the help of Tom20/Tom40 and Tim23 complex, SHP2 translocates into mitochondrial matrix and subsequently interacts with ANT1.

The key regulatory role of SHP2-ANT1 interplay in mitochondrial homeostasis and its involvement in inflammasome activation is supported by the robust rescuing effect of ANT1 inhibition in SHP2 knockdown cells (Fig. 8a,b). Furthermore, the Y191F mutant of ANT1 mimicked the effect of ANT1 knockdown in maintaining the mitochondrial integrity (Fig. 9d). Consistent with previous study, our findings emphasize the importance of N-SH2 domain in directing SHP2 to the appropriate subcellular location, mediating the binding of SHP2 to other signaling proteins, and determining the specificity of substrate interactions^42^.

The role of SHP2 has been established in various biological processes and diseases. Genetically, loss- and gain-of-function mutations in SHP2-encoding gene *PTPN11* have been identified in Noonan and LEOPARD syndromes^43^. *PTPN11* was first identified as a proto-oncogene, due to the activating mutations found in leukemia^44^. However, loss of SHP2/*PTPN11* promotes hepatocellular carcinoma^45^, suggesting that *PTPN11* also functions as a tumor suppressor. These opposing functions of the same gene are dependent on cellular context^46^. Given that innate immune response and inflammation are closely implicated in these diseases, the mechanisms we proposed may also contribute to the development of these pathological conditions. To conclude, we reveal a previously unknown regulation of SHP2 in NLRP3 inflammasome activation in the macrophages. Such negative regulation of SHP2, through dephosphorylating ANT1, maintains mitochondrial integrity and prevents excessive activation of inflammasome and the ensuing inflammation. Hence, fostering SHP2-ANT1-mediated mitochondrial homeostasis may offer a novel therapeutic approach for inducing resolution of NLRP3 inflammasome-dependent inflammatory diseases.

## Methods

### Chemicals, reagents and antibodies

Phorbolmyristate acetate (PMA, P1585), 4′,6-diamidino-2-phenylindole (D8417), LPS (L2630), ATP (A7699), Nigericin sodium salt (72445), MSU (U2875), and Ac-YVAD-cmk (SML0429) were purchased from Sigma-Aldrich (St. Louis, MO). The SHP2 inhibitor NSC-87877 and PHPS1 were purchased from Calbiochem (La Jolla, CA). NAC (S0077) was purchased from Beyotime (Nantong, China). Disuccinimidylsuberate (21655) was bought from Thermo  Fisher Scientific; enzyme-linked immunosorbent assay (ELISA) kits for murine or human IL-1β were purchased from Dakewe (Beijing, China). ELISA kits for murine or human IL-18 were purchased from Raybiotech (Norcross, GA). Anti-myc-tag (2276, 1 : 1,000 dilution) and anti-HA-tag (3724, 1 : 1,000 dilution) were purchased from Cell Signaling Technology (Beverly, MA). Anti-NLRP3 (ab17267, 1 : 1,000 dilution), anti-caspase-1 (ab108362, 1 : 1,000 dilution), anti-ANT1 (ab110322, 1 : 1,000 dilution), and anti-p-SHP2 (ab62322, 1 : 500 dilution) were purchased from Abcam (Cambridge, UK). Anti-PYCARD (ASC, sc-271054, 1 : 2,000 dilution), anti-Tom20 (sc-136211, 1 : 2,000 dilution), anti-Tom40 (sc-365467, 1 : 1,000 dilution), anti-Tom70 (sc-390545, 1 : 1,000 dilution), anti-Tim23 (sc-514463, 1 : 1,000 dilution), anti-HSP60 (sc-376240, 1 : 500 dilution), COX IV (sc-69359, 1 : 400 dilution), and anti-SHP2 (sc-7384, 1 : 500 dilution) were purchased from Santa Cruz Biotechnology (Santa Cruz, CA). Anti-Tim22 (14927-1-AP, 1 : 1,000 dilution) was purchased from Proteintech Group (Wuhan, China). Anti-Actin (M20010, 1 : 2,000 dilution) was purchased from Abmart (Shanghai, China). Anti-mouse Gr1-PE (12-593, dilution 1 : 50) and anti-mouse CD11b-APC (17-0112, dilution 1 : 50) were purchased for eBioscience (USA). JC-1 (T-3168), MitoSOX Red Mitochondrial Superoxide Indicator (M36008), Alexa Fluor 488 goat anti-rabbit IgG (A11008), Alexa Fluor 488 Donkey Anti-Goat IgG (A11055), Alexa Fluor 594 Goat Anti-Mouse IgG (A11032), and mitochondrial specific dye MitoTracker Red CMXRos (M7512) were purchased from Thermo Fisher Scientific  (MA, USA) Mitochondria/Cytosol Fractionation Kit (ab65320) was purchased from Abcam. Incomplete Freund’s adjuvant was purchased from Sigma-Aldrich. All other chemicals were obtained from Sigma-Aldrich.

### Plasmids and lentivirus

pET21b-caspase-1-His (Plasmid 11809), pCI-ASC-HA (Plasmid 41553), pGEX-4T1 SHP2 WT (Plasmid 8322), and pCMV-SHP2 (Plasmid 8381) were purchased from Addgene. Recombinant vectors encoding human ANT1, human pro-IL-1β, and human NLRP3 were constructed by PCR-based amplification of complementary DNA from THP-1 cells, and then were subcloned into the pcDNA3.1 eukaryotic expression vector. pCMV-SHP2-HA, pCMV-SHP2-D61A-HA, pCMV-SHP2-C459S-HA, SHP2-mut-HA (R4R5A4A5), SHP2-ΔPTP-HA, SHP2-ΔSHP2-HA, ANT1-Y191F-myc and ANT1-Y195F-myc were obtained by PCR-based mutation and amplification of WT expression vector. Plasmids were transiently transfected into HEK293T cells. The shRNA-Tim22 (sc-94220-V), shRNA-Tim23 (sc-44155-V) and shRNA-Ctrl (sc-108080) were purchased from Santa Cruz Biotechnology. The lentivirus for shRNA-SHP2, shRNA-ANT1‚ shRNA-Tom20, shRNA-Tom40, shRNA-Tom70, and shRNA-scramble (shRNA-Ctrl) were purchased from Shanghai Obio Technology Co. Ltd. (Shanghai, China). The sequences were 5′-TTCTCCGAACGTGTCACGT-3′ (shRNA-Ctrl), 5′-ACACTGGTGATTACTATGA-3′ (shRNA-SHP2), 5′-CCTTTGACACTGTTCGTCGTA-3′ (shRNA-ANT1), 5′-GCTCACTTTCCCTCCATTT-3′ (shRNA-Tom20), 5′-GCAAGAACAAGTTTCAGTG-3′ (shRNA-Tom40), and 5′-GCATGCTGTTAGCCGATAA-3′ (shRNA-Tom70), respectively.

### Generation of cSHP2-KO mice

The macrophage-specific cSHP2-KO mice were generated by crossing SHP2^flox/flox^ mice with Lyz2-Cre transgenic mice (Supplementary Fig. 1). The animals were maintained with free access to pellet food and water in plastic cages at 21 ± 2 °C and kept on a 12 h light–dark cycle. All mice are in C57BL/6 background and are harbored in the specific pathogen-free facility in Nanjing University. Eight-week-old female cSHP2-KO mice and WT littermates were used. Animal welfare and experimental procedures were carried out in accordance with the Guide for the Care and Use of Laboratory Animals (National Institutes of Health, USA) and the related ethical regulations of our university. All efforts were made to reduce the number of animals used and to minimize animal suffering.

### Cell culture

Human monocytic THP-1 cell line was purchased from Shanghai Institute of Cell Biology (Shanghai, China) and cultured at 37 °C in a 5% (v/v) CO_2_ atmosphere. Before further stimulation, THP-1 cells were treated with PMA (500 nM) for 12 h. Peritoneal macrophages were harvested from mice by flushing the peritoneal cavity with 5 ml ice-cold phosphate-buffered saline (PBS). Cells were then centrifuged at 300 *g* for 10 min and allowed to adhere to glass coverslips overnight. Non-adherent cells were washed away with PBS and attached cells were maintained in culture. Bone marrow-derived macrophages were isolated from C57BL/6 mice and cultured with Dulbecco's modified Eagle's medium supplemented with 10% fetal bovine serum and 20 ng ml^−1^ recombinant murine macrophage colony-stimulating factor (PeproTech, 315-02). Culture fluid was exchanged with fresh culture medium every 3 day. Under these conditions, an adherent macrophage monolayer was obtained at day 7.

### Immunoblot assay

Immunoblot assay was performed as described previously^47^. Briefly, proteins were extracted in lysis buffer. The proteins were then separated by SDS–polyacrylamide gel electrophoresis (PAGE) and electrophoretically transferred onto polyvinylidene difluoride membranes. The membranes were probed with antibodies overnight at 4 °C, and then incubated with a horseradish peroxidase-coupled secondary antibody. Detection was performed using a LumiGLO chemiluminescent substrate system. Full-length uncropped blots are presented in Supplementary Figures 10–16.

### Co-immunoprecipitation assay

Proteins from cells were incubated with 1 μg of appropriate antibody and precipitated with protein A/G-agarose beads (Santa Cruz Biotechnology). The immunoprecipitated proteins were separated by SDS–PAGE and immunoblot was performed with the indicated antibodies.

### The quantification of mtDNA by quantitative PCR

The quantification of mtDNA was performed as described previously^5^. Total DNA was isolated from cells with a DNeasy Blood & Tissue kit (Qiagen). For the extraction 1 × 10^7^ cells were homogenized with a Dounce homogenizer in 100 mM Tricine-NaOH solution, pH 7.4, containing 0.25 M sucrose, 1 mM EDTA, and protease inhibitor, then were centrifuged at 700 *g* for 10 min at 4 °C. Protein concentration and volume of the supernatant were normalized, followed by centrifugation at 10,000 *g* for 30 min at 4 °C for the production of a supernatant corresponding to the cytosolic fraction. DNA was isolated from 200 µl of the cytosolic fraction. Quantitative PCR was performed on BioRadCFX96 Touch Real-Time PCR Detection System (Bio-Rad) by using iQ SYBR Green Supermix (1708880, Bio-Rad), and threshold cycle numbers were obtained using BioRad CFX Manager software. The program for amplification was 1 cycle of 95 °C for 2 min followed by 40 cycles of 95 °C for 10 s, 60 °C for 30 s, and 95 °C for 10 s. The copy number of mtDNA was normalized to nuclear DNA (cytochrome *c* oxidase I/18S ribosomal RNA). The primers sequence was provided in the Supplementary Table 2.

### ASC pyroptosome detection

ASC pyroptosomes were detected as described previously^48^. THP-1 cells were pelleted by centrifugation and resuspended in 0.5 ml of ice-cold buffer containing 20 mM HEPES-KOH, pH 7.5, 150 mM KCl, 1% Nonidet P-40, 0.1 mM phenylmethylsulfonyl fluoride and a protease inhibitor mixture, and lysed by shearing 10 times through a 21-gauge needle. The cell lysates were then centrifuged at 5000 *g* for 10 min at 4 °C, and the resultant pellets were washed twice with PBS and resuspended in 500 µl of PBS. Next, the resuspended pellets were cross-linked with fresh disuccinimidylsuberate (4 mM) for 30 min and pelleted by centrifugation at 5000 *g* for 10 min. The cross-linked pellets were resuspended in 30 µl of SDS sample buffer separated using 12% SDS–PAGE and immunoblotted using anti-mouse ASC antibodies.

### Alum-induced peritonitis in mice

Eight-week-old female C57BL/6 mice (10 mice per group) were intraperitoneally (i.p.) injected with 700 mg Alum (Thermo  Fisher Scientific) as described previously^24^. For analysis of inflammatory cell subsets, mice were killed 12 h after Alum injection and peritoneal cavities were washed with 6 ml of PBS. PECs were collected and analyzed by flow cytometry. The numbers of neutrophils and monocytes in each mouse were calculated according to its proportion in PECs (Supplementary Fig. 2). For the analysis of IL-1β in the peritoneal cavity, 8 h after i.p. injection of Alum, peritoneal cavities were washed with cold PBS. Then the peritoneal fluids were concentrated for ELISA analysis.

### Preparation of the subcellular fractions

The cytoplasmic and mitochondrial fractions were prepared by using the Mitochondria Isolation Kit (Thermo Fisher Scientific) according to the manufacturer’s instructions. Briefly, THP-1 cells were lysed by reagents A, B, and C supplied with the kit and centrifuged at 700 *g* at 4 °C for 10 min to obtain a postnuclear upernatant. The mitochondria were pelleted by centrifugation at 10,000 *g* at 4 °C for 15 min. The supernatant fraction was the cytosolic protein fraction. The various fractions were analyzed by SDS–PAGE. For protease digestion, fractions of mitochondria (resuspended in 20 mM HEPES-KOH, pH 7.4, 250 mM sucrose, 80 mM KOAc, and 5 mM MgOAc) were incubated with 40 μM proteinase K (Sigma-Aldrich) for 30 min on ice. Digestion was stopped with 1 mM PMSF and the samples were determined by immunoblot analysis. Alkaline extraction was carried out as previously reported^21^. Briefly, mitochondrial samples were lysed in 0.1 M Na_2_CO_3_, pH 11.5, on ice for 30 min with occasional vortexing. The membranes were isolated by centrifugation at 100,000 *g* for 30 min at 4 °C and analyzed by immunoblot analysis.

### Statistical analysis

Data are expressed as mean ± SEM. Statistically evaluated by Student’s *t*-test when only two value sets were compared and one-way analysis of variance (ANOVA) followed by Dunnett’s test when the data involved three or more groups. *P* < 0.05 was considered significant.

### Data availability

The data that support this study are available within the article and its Supplementary Information files or available from the authors upon request.

## Electronic supplementary material

Supplementary information

Description of Additional Supplementary Files

Supplementary Movie 1

Supplementary Movie 2
