# Supplementary material for: Tyrosine phosphatase SHP2 negatively regulates NLRP3 inflammasome activation via ANT1-dependent mitochondrial homeostasis
Source: Nat Commun. 2017 Dec 18;8:2168. doi: 10.1038/s41467-017-02351-0 (PMC5735095; doi:10.1038/s41467-017-02351-0)
Supplement: Supplementary file 1 — Supplementary information [file 41467_2017_2351_MOESM1_ESM.pdf]

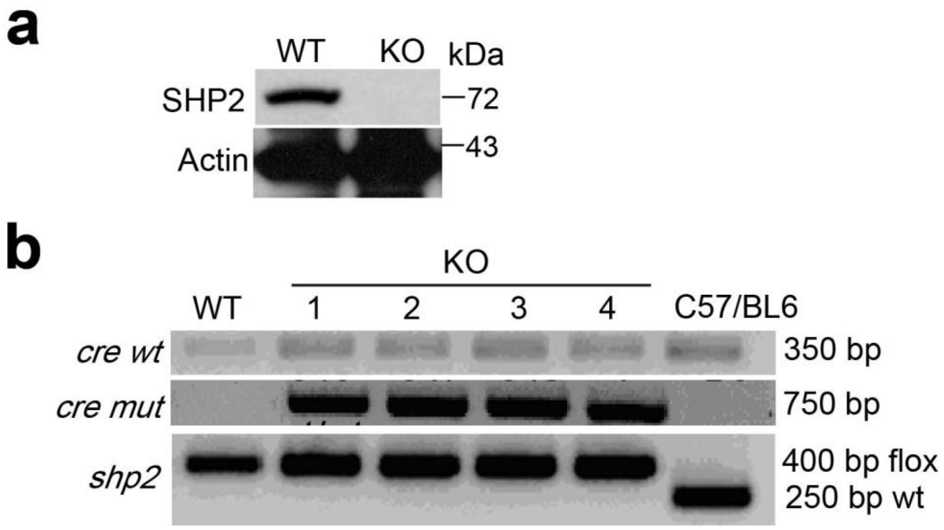

Supplementary Fig. 1. Identification of macrophage-specific conditional SHP2 knockout mice. (**a**) Immunoblot analysis of the expression of SHP2 in F4/80$^+$ peritoneal macrophage cells from WT and conditional SHP2 knockout (KO) mice. (**b**) PCR analysis of tail samples from indicated mice. Data are representative of three independent experiments.

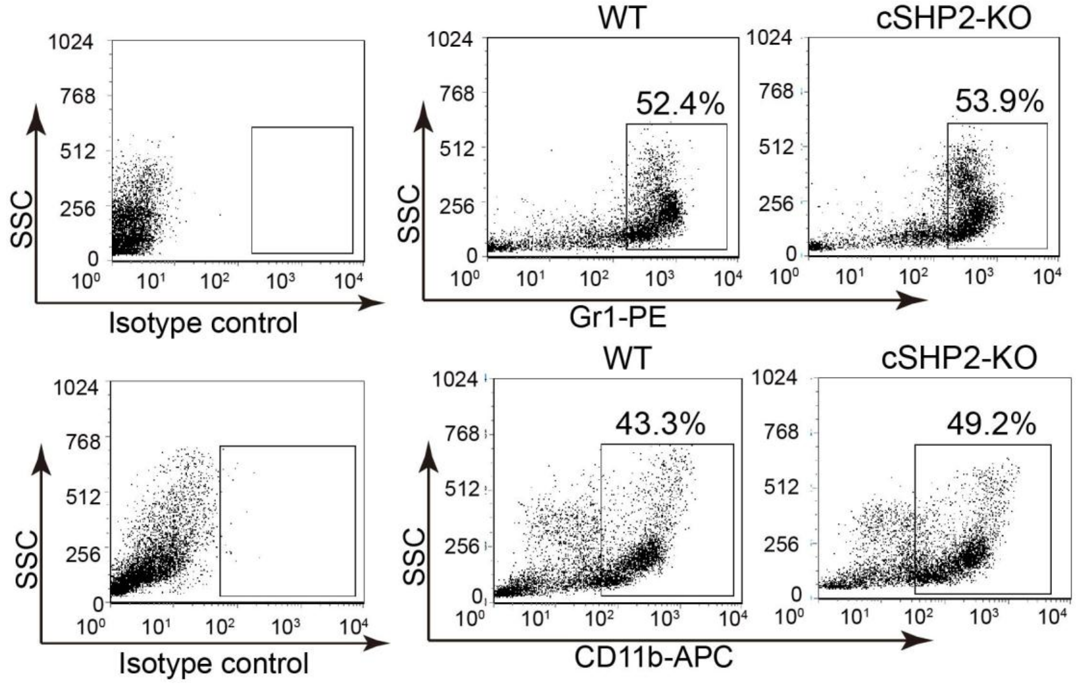

Supplementary Fig. 2. Flow cytometry analysis of peritoneal exudate cells 12 h after Alum injection in mice. Eight-week-old female C57BL/6 mice (10 mice per group) were i.p. injected with 700 mg Alum. For analysis of inflammatory cell subsets, mice were sacrificed 12 h after Alum injection and peritoneal cavities were washed with 6 ml of PBS. Peritoneal exudate cells (PECs) were collected and stained with Gr1-PE (neutrophils) and CD11b-APC (monocytes), respectively and analyzed by flow cytometry. Isotype control was used for gating. The numbers of neutrophils and monocytes in each mouse were calculated according to its proportion in PECs. Representative flow cytometry data from WT and cSHP2-KO mice are shown.

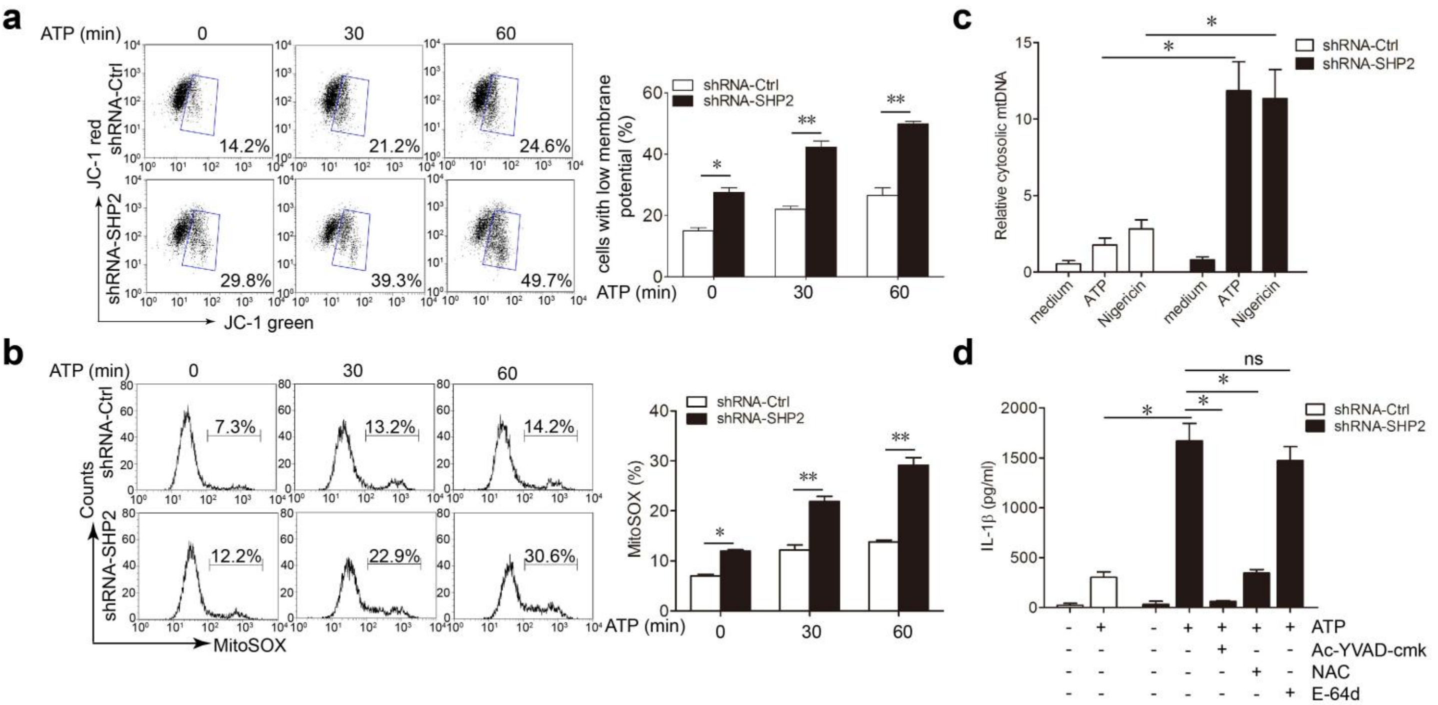

Supplementary Fig. 3. SHP2 knockdown leads to mitochondrial dysfunction and excessive NLRP3 inflammasome activation. (**a**,**b**) Flow cytometry analysis of mitochondrial membrane potential by JC-1 staining (**a**) or mitochondrial ROS by MitoSOX staining (**b**) from SHP2-knockdown THP-1-derived macrophages, and left untreated or treated with ATP (5 mM) for indicated times. (**c**) Quantitative real-time PCR analysis of mtDNA released from SHP2-knockdown THP-1-derived macrophages and left untreated (medium) or primed with LPS (100 ng/ml) for 3 h and stimulated with ATP (5 mM, 1 h) and Nigericin (10 μM, 2 h). (**d**) ELISA of IL-1β in supernatants of SHP2-knockdown THP-1-derived macrophages, which were primed with LPS (100 ng/ml) for 3 h, and left untreated or treated with Ac-YVAD-cmk (30 μM), NAC (5 mM) or E-64d (20 μM) for 1 h, followed by stimulation of ATP (5 mM) for 1 h. **P*<0.05, ***P*<0.01, one-way ANOVA for multiple comparisons, ns represents no significance. Data are presented as mean ± s.e.m. of three independent experiments (**a-d**).

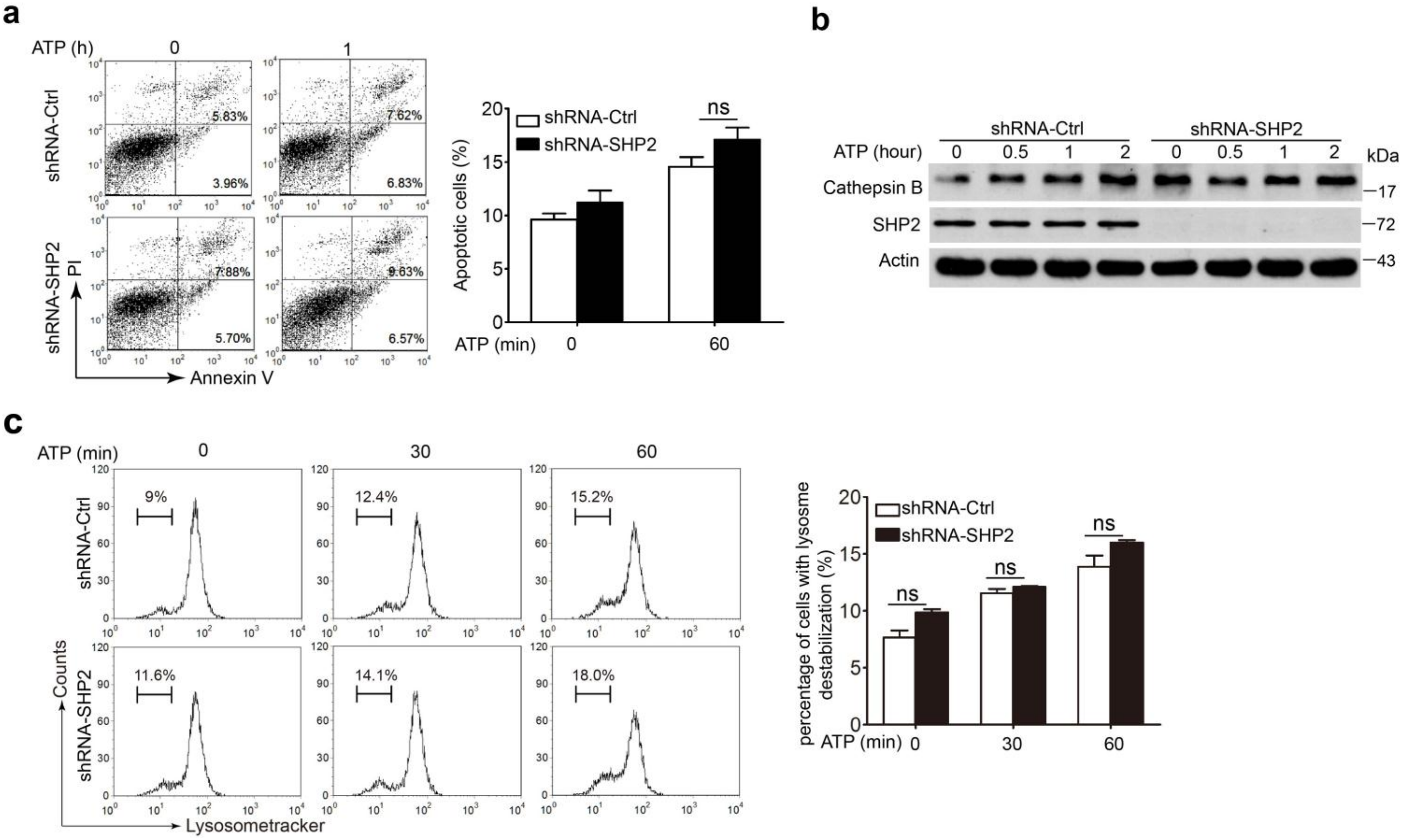

Supplementary Fig. 4. SHP2 does not affect cell survival and lysosome stabilization during ATP treatment. (**a**) Flow cytometry analysis of apoptosis by Annexin V/PI staining in SHP2-knockdown THP-1-derived macrophages, and left untreated or treated with ATP (5 mM) for indicated times. (**b**) Immunoblot analysis of the expression of Cathepsin B in SHP2-knockdown THP-1-derived macrophages, and left untreated or treated with ATP (5 mM) for indicated times. (**c**) Flow cytometry analysis of lysosome stabilization by lysosome tracker staining (LysoTracker Red DND-99, L7528, Invitrogen, Carlsbad, CA) in SHP2-knockdown THP-1-derived macrophages, and left untreated or treated with ATP (5 mM) for indicated times. ns represents no significance, one-way ANOVA for multiple comparisons. Data are representative of three independent experiments (mean and s.e.m. of three independent samples in **a**, **c**).

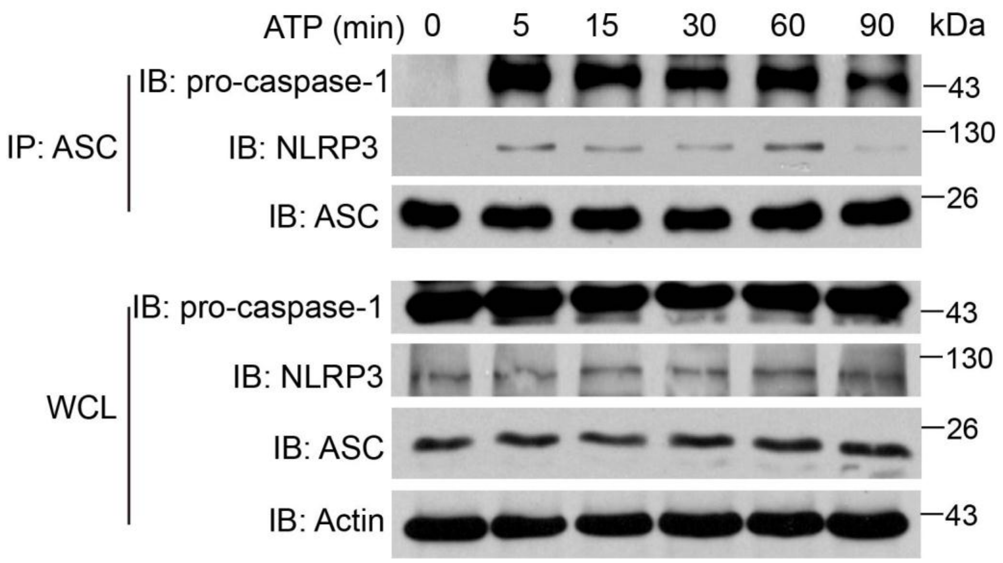

Supplementary Fig. 5. Time course for NLRP3 inflammasome assembly upon ATP stimulation. Co-immunoprecipitation (Co-IP) analysis of the NLRP3 inflammasome components in THP-1-derived macrophages, and left untreated (medium) or stimulated with 5 mM ATP for indicated times. Data are representative of three independent experiments.

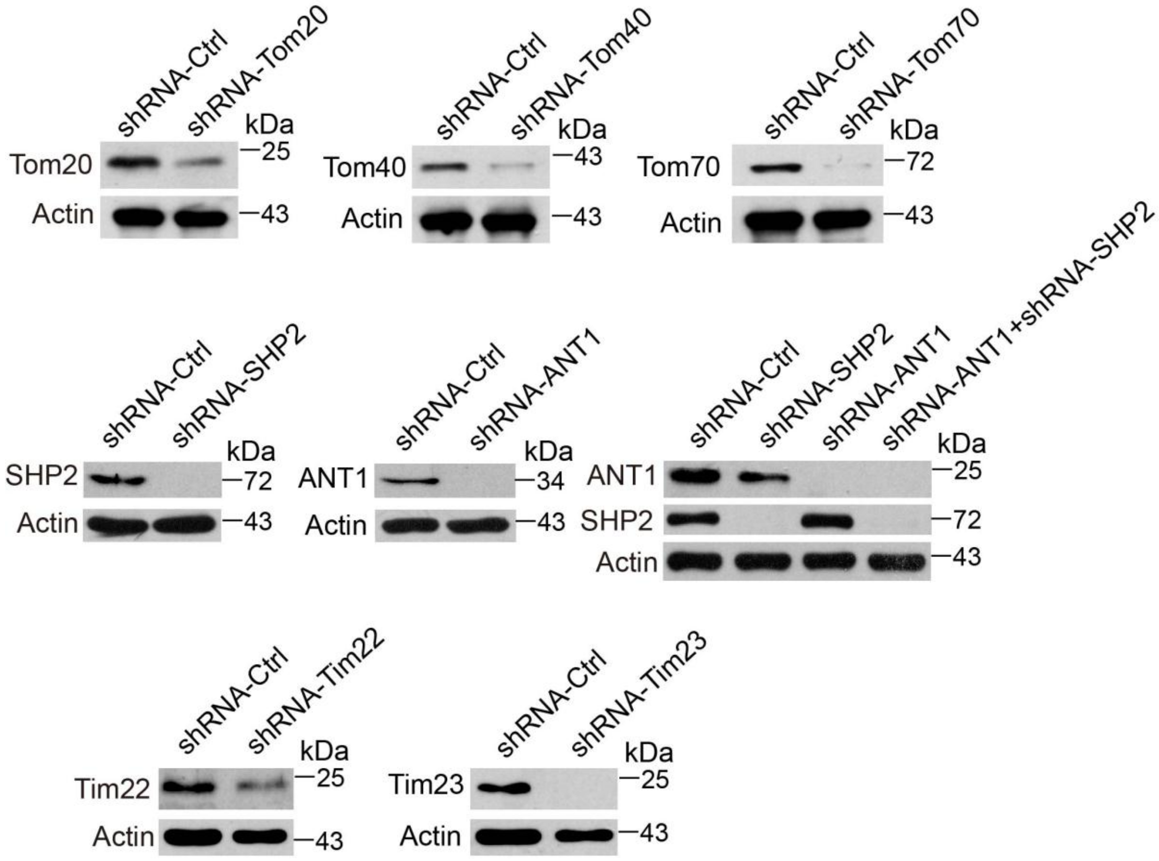

Supplementary Fig. 6. Generation of various genes knockdown THP-1 cells. Immunoblot analysis of indicated protein in THP-1 cells which were transfected with various lentivirus for 3 days. In the present study, a dose of 50 IU shRNA-Tom20, shRNA-Tom40, shRNA-Tom70, shRNA-SHP2, shRNA-ANT1 or shRNA-Ctrl (shRNA-scramble) lentivirus (Obio Technology Co. Ltd., Shanghai, China), shRNA-Tim22, shRNA-Tim23 or shRNA-Ctrl (Santa Cruz Biotechnology, Santa Cruz, CA) per cell was used to infect THP-1 cells. After 3 days of infection, THP-1 cells were used in the further experiments. Data are representative of three independent experiments.

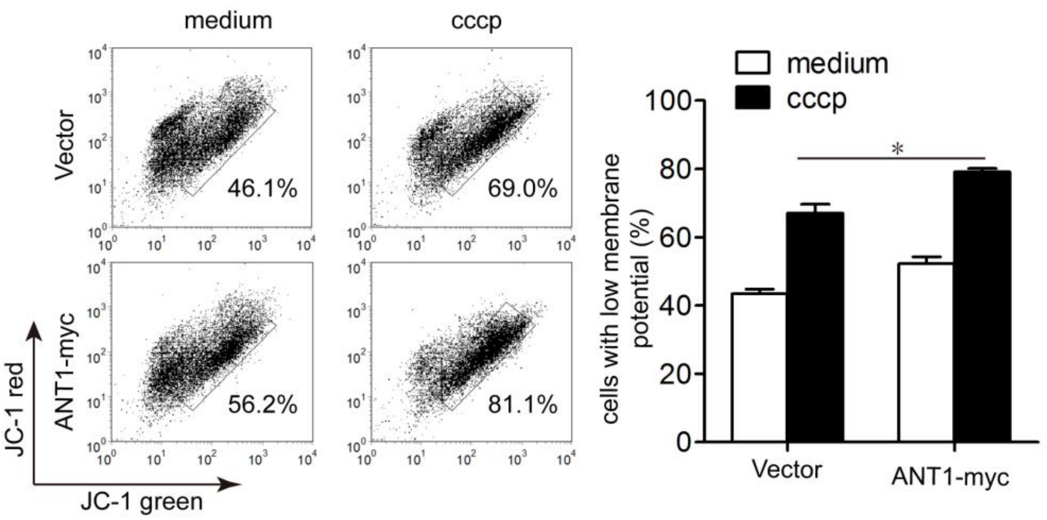

Supplementary Fig. 7. ANT1 overexpression promotes permeabilizaion of mitochondria. Flow cytometry analysis of mitochondrial membrane potential by JC-1 staining in HEK293T cells which were transfected with ANT1-myc plasmid followed by cccp treatment (20 μM, 1 h). **P*<0.05 by Student's *t*-test. Data are presented as means ± s.e.m. of three independent experiments.

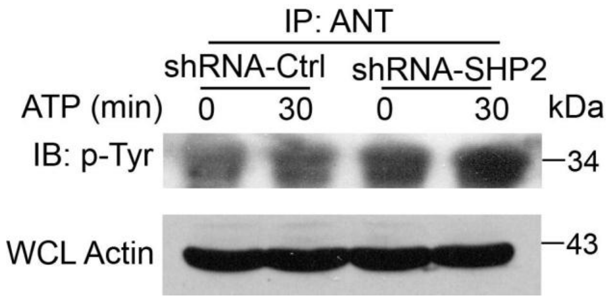

Supplementary Fig. 8. ANT1 is phosphorylated under ATP stimulation. Co-immunoprecipitation (Co-IP) analysis of ANT1 tyrosine phosphorylation in SHP2-knockdown THP-1-derived macrophages upon ATP treatment. THP-1 cells, which were transfected with shRNA-SHP2 or shRNA-Ctrl (shRNA-scramble) lentivirus for 3 days, were stimulated with 5 mM ATP for 30 min. Then the total Tyr phosphorylation level of immunoprecipitated ANT1 were examined by immunoblot analysis. Data are representative of three independent experiments.

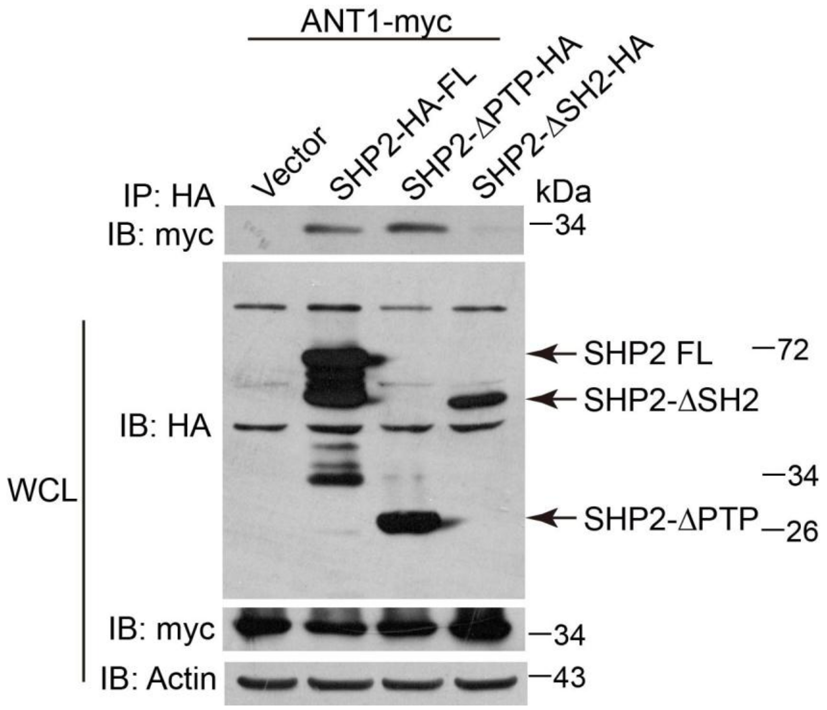

Supplementary Fig. 9. The identification of interaction domain between SHP2 and ANT1. Co-immunoprecipitation (Co-IP) analysis of interaction domain between SHP2 and ANT1 in HEK293T cells. HEK293T cells were transfected with ANT1-myc and SHP2-HA-FL or SHP2-ΔPTP-HA or SHP2-ΔSH2-HA plasmid, respectively and subjected to Co-IP assay. Data shown are representative of three independent experiments.

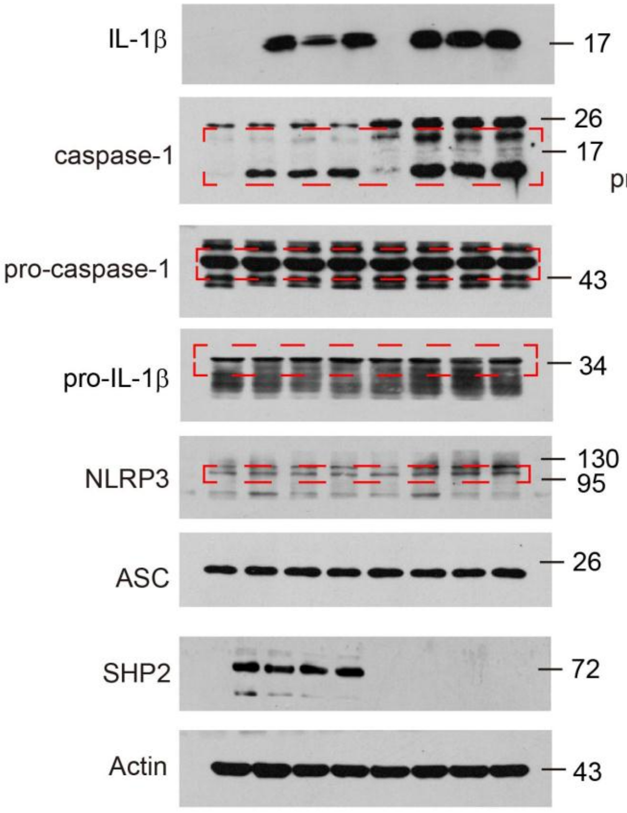

Fig. 1c

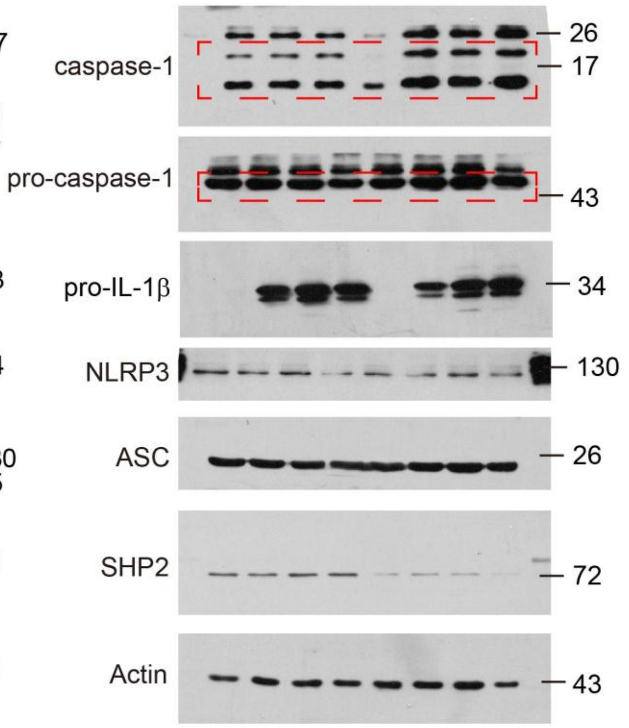

Fig. 1f

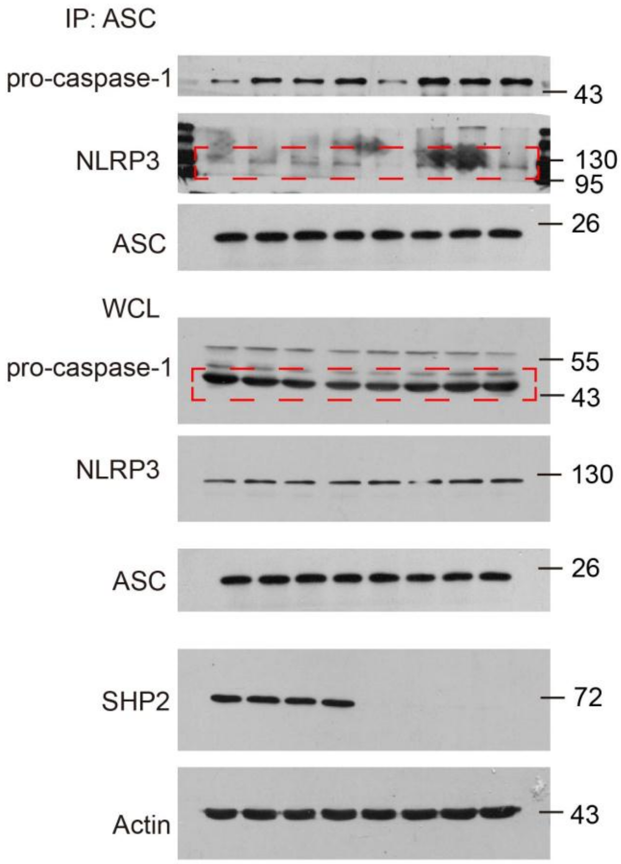

Fig. 1h

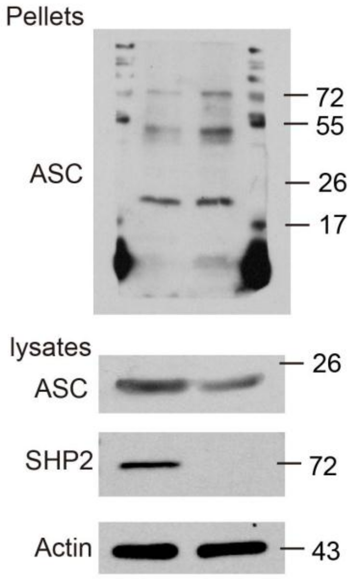

Fig. 1i

Supplementary Fig. 10. Full scans of original blots for data in Figure 1. Panels corresponding to the figures in the manuscript are indicated.

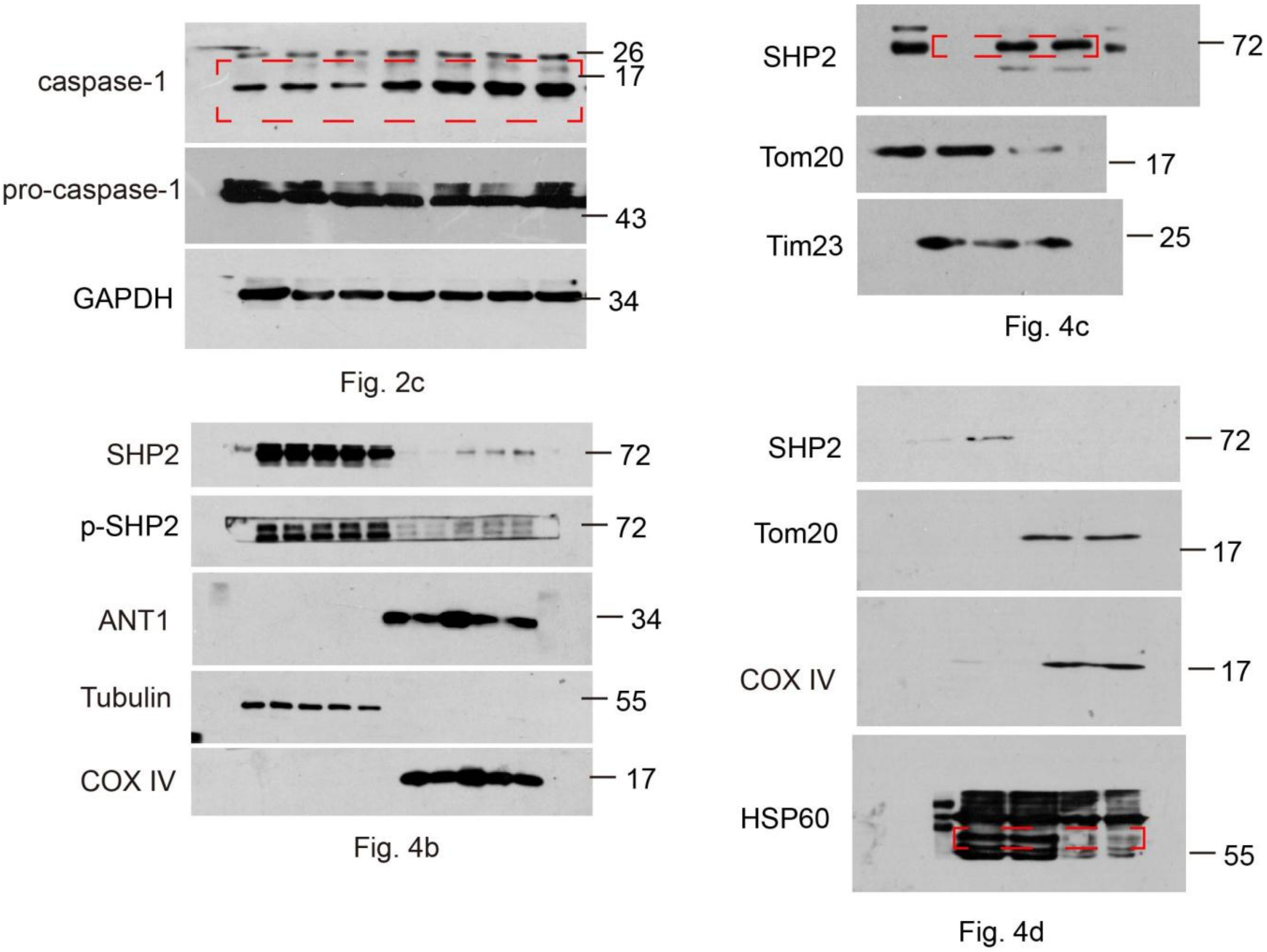

Supplementary Fig. 11. Full scans of original blots for data in Figures 2 and 4. Panels corresponding to the figures in the manuscript are indicated.

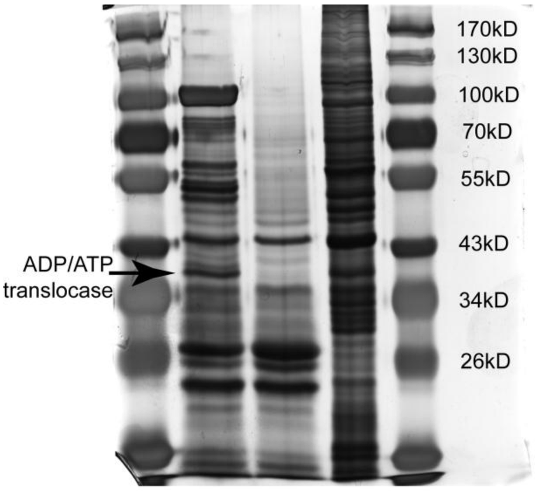

Fig. 5a

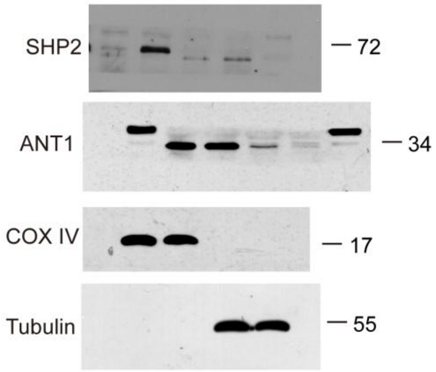

Fig. 5d

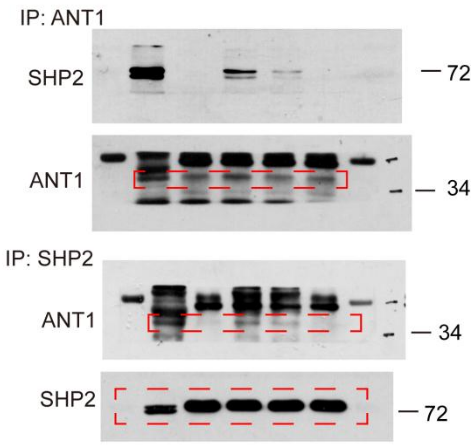

Fig. 5c

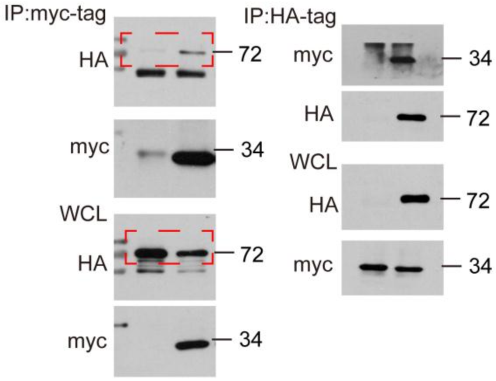

Fig. 5e

Supplementary Fig. 12. Full scans of original blots for data in Figure 5. Panels corresponding to the figures in the manuscript are indicated.

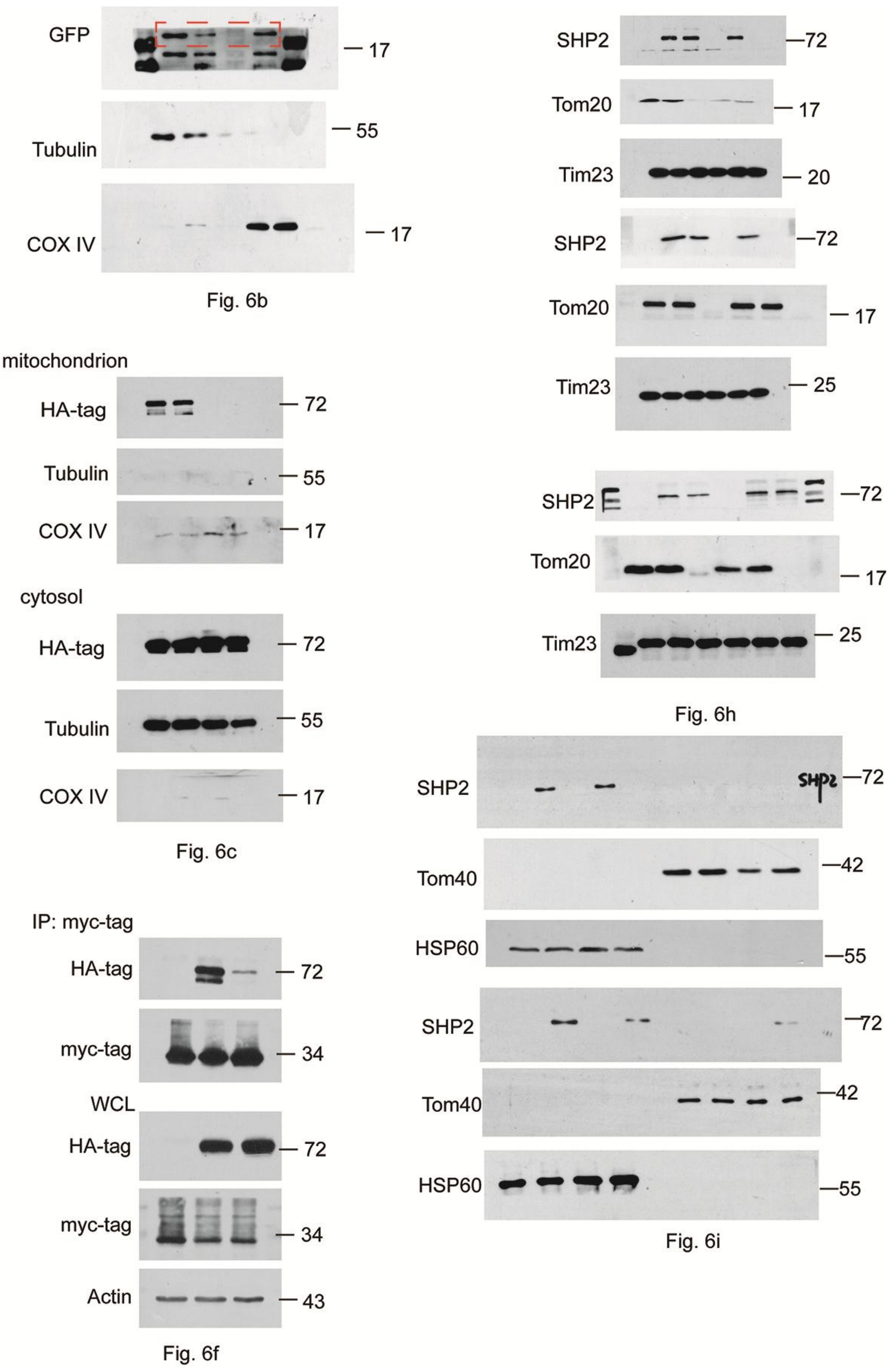

Supplementary Fig. 13. Full scans of original blots for data in Figure 6. Panels corresponding to the figures in the manuscript are indicated.

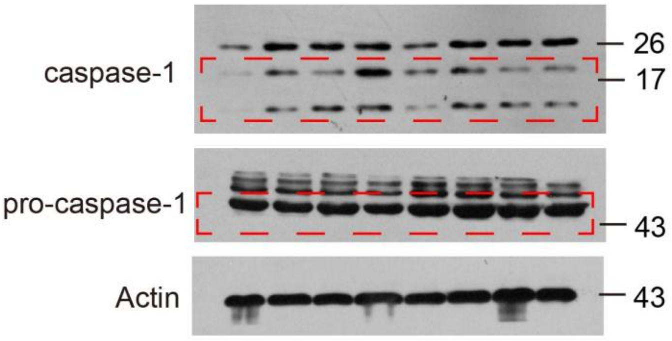

Fig. 7c

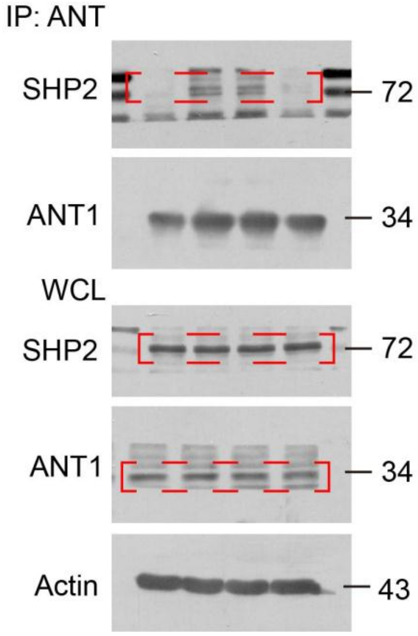

Fig. 8d

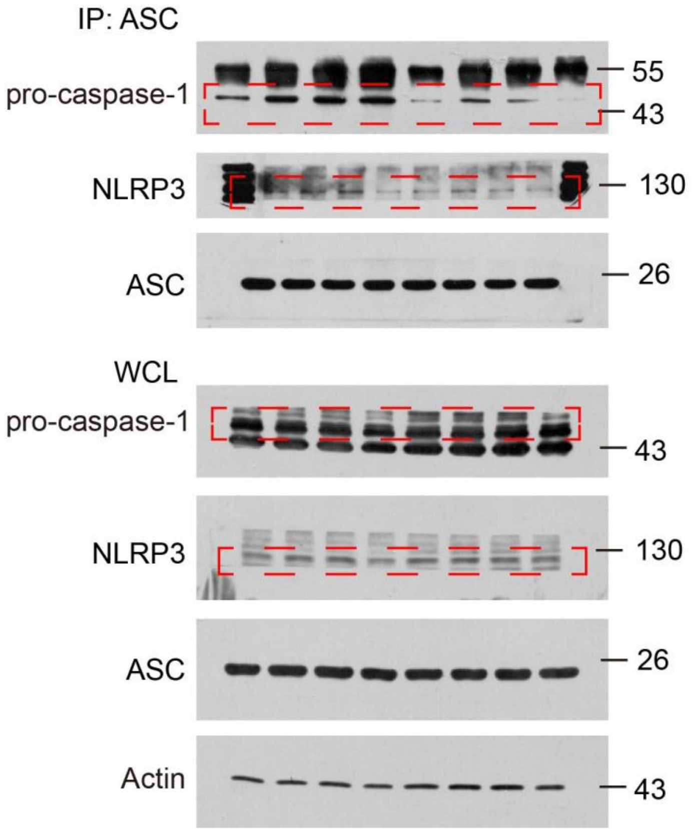

Fig. 7d

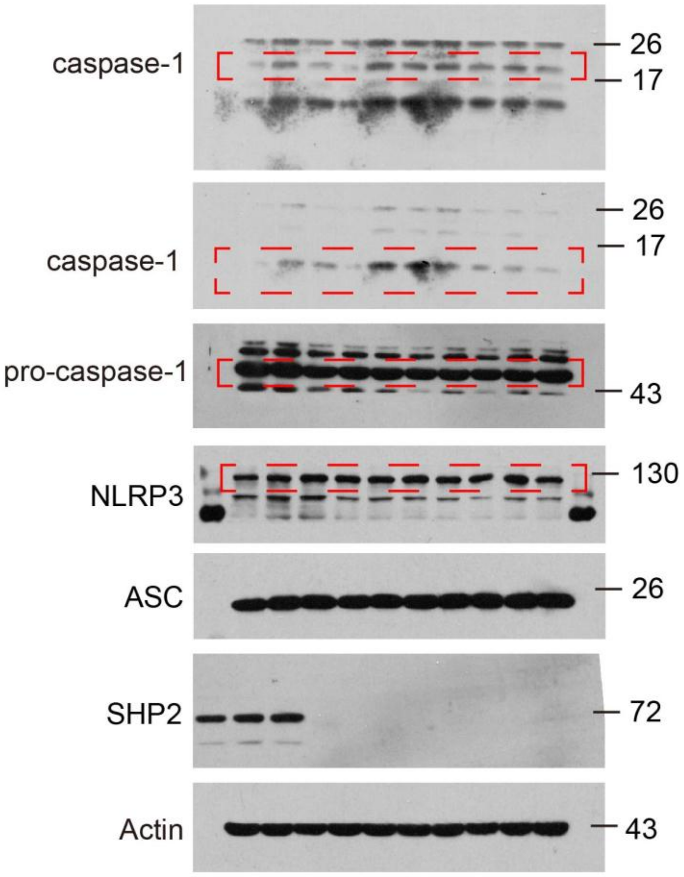

Fig. 8f

Supplementary Fig. 14. Full scans of original blots for data in Figures 7 and 8. Panels corresponding to the figures in the manuscript are indicated.

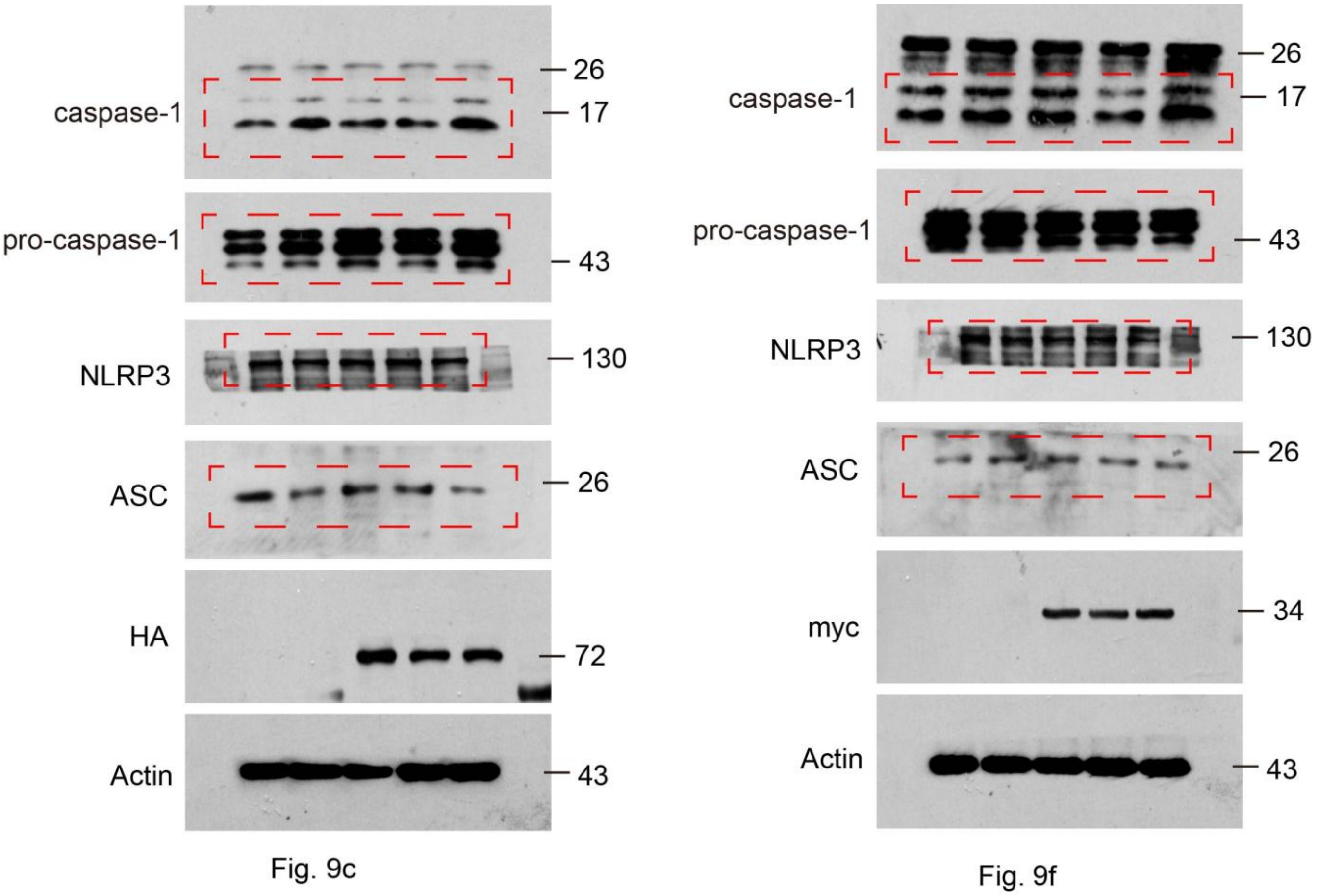

Supplementary Fig. 15. Full scans of original blots for data in Figure 9. Panels corresponding to the figures in the manuscript are indicated.

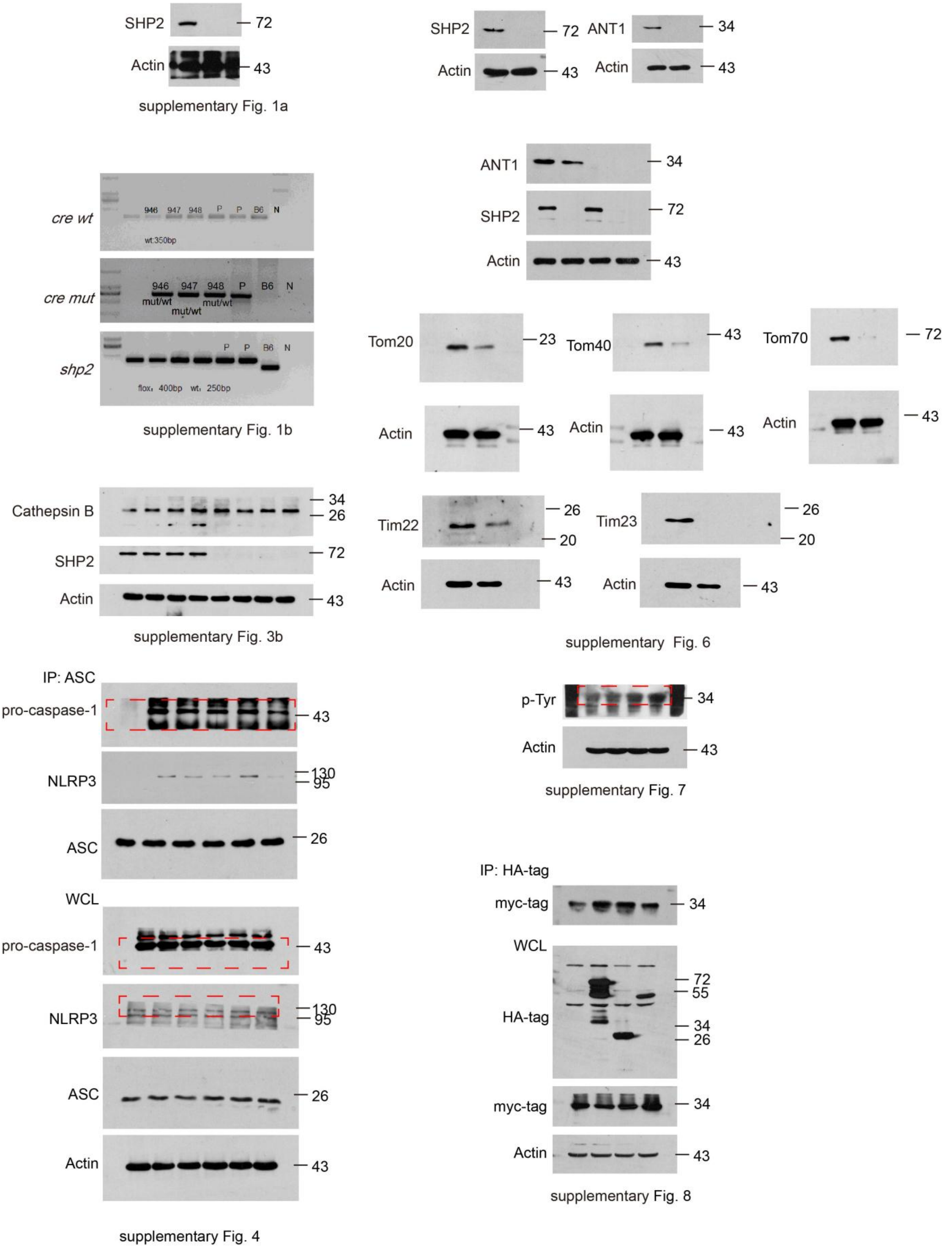

Supplementary Fig. 16. Full scans of original blots for data in Supplementary Figures. Panels corresponding to the figures in the manuscript are indicated.

Supplementary Table 1. Determination of GST-SHP2-pulldowned protein by liquid chromatography coupled with mass spectrometry (LC-MS).

Protein view	Mass	Score	Expect	Matches	protein name
gi|62089230	35589	227	4.90E-18	17	ADP,ATP carrier protein, liver isoform T2 variant
gi|156071462	33073	175	7.80E-13	15	ADP/ATP translocase 3
gi|15928608	33133	154	9.80E-11	14	Solute carrier family 25 (mitochondrial carrier; adenine nucleotide translocator), member 6
gi|339723	29331	118	3.90E-07	10	ADP.ATP translocase, partial
gi|158257236	33287	96	5.50E-05	9	unnamed protein product
gi|55749577	33271	96	5.50E-05	9	ADP/ATP translocase 1
gi|156071459	33059	96	7e-05	10	ADP/ATP translocase 2
gi|339920	33355	95	7.80E-05	9	ADP/ADT translocator protein
gi|45829841	35499	95	8.00E-05	10	SLC25A5 protein, partial
gi|388452552	33102	94	9.40E-05	10	ADP/ATP translocase 2
gi|339721	28271	80	0.0028	8	ADP.ATP translocase, partial
gi|189054857	33062	79	0.003	8	unnamed protein product
gi|179247	33188	78	0.0038	9	ADP/ATP carrier protein
gi|119610275	27843	70	0.025	8	solute carrier family 25 (mitochondrial carrier; adenine nucleotide translocator), member 5, isoform CRA_b
gi|119610783	37167	50	2.5	6	enolase 3 (beta, muscle), isoform CRA_c
gi|194380266	15857	49	3.3	5	unnamed protein product
gi|194386838	116274	47	4.7	9	unnamed protein product
gi|400530116	31743	42	17	6	phosphoinositide-3-kinase regulatory subunit, partial
gi|400530118	31929	41	18	6	phosphoinositide-3-kinase regulatory subunit, partial
gi|221040434	33229	39	30	6	unnamed protein product
gi|163931057	33647	38	36	6	Chain B, Structure Of A Human P110alphaP85ALPHA COMPLEX
gi|8918269	8150	38	39	3	phosphodiesterase I/nucleotide pyrophosphatase 1
gi|193787728	43010	38	41	6	unnamed protein product
gi|335057534	43040	38	41	6	phosphatidylinositol 3-kinase regulatory subunit alpha isoform 4
gi|299689340	46005	37	47	6	Chain A, The Catalytic Domain Of Usp8 In Complex With A Usp8 Specific Inhibitor
gi|5442028	15462	37	50	3	aminopeptidase
gi|119626986	17063	36	61	3	hCG2038632
gi|32455248	83889	36	64	7	phosphatidylinositol 3-kinase regulatory subunit alpha isoform 1
gi|119583685	9938	36	67	4	hCG1808265
gi|343790880	34642	36	68	4	dehydrodolichyl diphosphate synthase

					isoform 4
gi|194381290	69632	42	15	7	unnamed protein product
gi|39777597	78420	40	26	7	protein-glutamine gamma-glutamyltransferase 2 isoform a
gi|390136164	79313	40	28	7	Chain A, Transglutaminase 2 In Complex With A Novel Inhibitor
gi|303324982	79717	40	28	7	Chain A, Crystal Structure Of Human Transglutaminase 2 Complex With Adenosine 5' Triphosphate

Supplementary Table 2. Sequences of PCR primer pairs used in the present study.

cytochrome *c* oxidase I for mouse mtDNA	S: 5'- GCCCCAGATATAGCATTCCC<br>AS: 5'- GTTCATCCTGTTCCTGCTCC
18S ribosomal RNA for mouse nDNA	S: 5'- TAGAGGGACAAGTGGCGTTC<br>AS: 5'- CGCTGAGCCAGTCAGTGT
cytochrome *c* oxidase I for human mtDNA	S: 5'- CTGTCTTTGATTCCTGCCTC<br>AS: 5'- TTGAGGAGGTAAGCTACAT
18S ribosomal RNA for Human nDNA	S: 5'- ATCATGTTTGAGACCTTCAACA<br>AS: 5'- CATCTCTTGCTCGAAGTCCA
