# Supplementary material for: Tyrosine phosphatase SHP2 negatively regulates NLRP3 inflammasome activation via ANT1-dependent mitochondrial homeostasis
Source: Nat Commun. 2017 Dec 18;8:2168. doi: 10.1038/s41467-017-02351-0 (PMC5735095; doi:10.1038/s41467-017-02351-0)
Supplement: Supplementary file 2 — Description of Additional Supplementary Files [file 41467_2017_2351_MOESM2_ESM.pdf]

Description of Additional Supplementary Files

File Name: Supplementary Movie 1
Description: **The location of SHP2 in mitochondria under physiological conditions.** 3D-reconstitution image of location of SHP2 (Red) and TOM20 (Green) in bone marrow-derived macrophages with medium treatment by structured-illumination microscopy.

File Name: Supplementary Movie 2
Description: **The location of SHP2 in mitochondria upon ATP treatment**. 3Dreconstitution image of location of SHP2 (Red) and TOM20 (Green) in bone marrow-derived macrophages with 5 mM ATP for 30 min by structured-illumination microscopy.
